# Motion-Controlled Photocatalytic Hydrogen Evolution Using Microrobots Designed with a Single Atomic-Level Precision

**DOI:** 10.1021/jacs.5c05661

**Published:** 2025-06-13

**Authors:** Anna Jancik-Prochazkova, Riku Nakao, Yuichi Yamaguchi, Akihiko Kudo, Katsuhiko Ariga

**Affiliations:** † Research Center for Materials Nanoarchitectonics, 52747 National Institute for Materials Science (NIMS), 1-1 Namiki, Tsukuba 305-0044, Japan; ‡ Department of Applied Chemistry, Faculty of Science, Tokyo University of Science, 1-3 Kagurazaka, Shinjuku-ku, Tokyo 162-8601, Japan; § Carbon Value Research Center, Research Institute for Science and Technology, Tokyo University of Science, 2641 Yamazaki Noda-Shi, Chiba-ken 278-8510, Japan; ∥ Graduate School of Frontier Sciences, The University of Tokyo, 515 Kashiwa no ha, Kashiwa 2778561, Japan

## Abstract

The potential of hydrogen as a next-generation fuel has recently attracted a great deal of attention because it is considered a green fuel originating from renewable sources. Material sciences with the tools of nanoarchitectonics are targeting a wide variety of suitable photocatalysts of different materials, morphologies, and dimensionalities. Here, we present the concept of the photocatalytic hydrogen evolution reaction (HER) using microrobots: tiny autonomous devices possessing propulsion and photocatalytic abilities. The microrobots were derived from a black TiO_2_ (bTiO_2_) material that provided the photocatalytic properties that contributed not only to successful light-induced propulsion but also to the activity toward the HER. In the next step, the decoration with magnetic nanoparticles (NPs) enabled the navigation of microrobots (mag-bTiO_2_ microrobots) in a magnetic field to enhance overall propulsion abilities and to allow their collection and consecutive reusability. As a result, mag-bTiO_2_ microrobots showed efficiency as dynamic photocatalysts for the HER; the positive contribution of the “on-the-fly” mode was confirmed by a control experiment using mag-bTiO_2_ microrobots as static photocatalysts. Furthermore, the overall efficiency of the HER was improved by decorating microrobots with atomic-level Pt species (mag-Pt-bTiO_2_ microrobots). The findings of this proof-of-concept study demonstrate an alternative approach toward the photocatalytic HER and lay the basis for the next generation of nano/microrobots for energy conversion applications.

## Graphical abstract

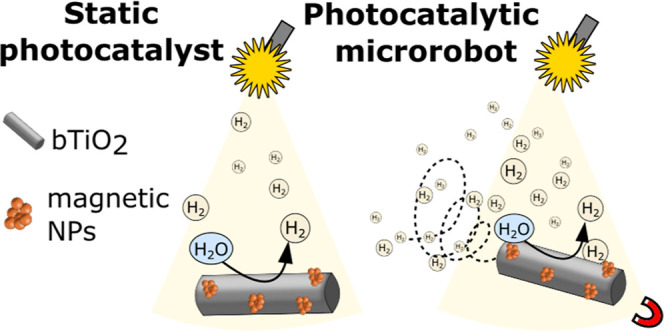

## Introduction

The need for sustainable solutions in energetics is palpable.^1^ The community is aware of the need to explore green sources of sustainable energy supply; there have been many different routes to find the applicable solutions.^2^ One of the routes would be the photocatalytic hydrogen evolution reaction (HER), which results from water splitting and thus provides hydrogen (H_2_) as a fuel that originates from renewable sources.^3,4^ Considering the photocatalytic HER as a sustainable and green source for the generation of H_2_, different aspects must be evaluated, especially the accessibility of technologies, the overall economics, safety, and environmental aspects, among others.^5^ Materials science is targeting a class of suitable photocatalysts that meet all critical aspects: not only efficiency but also sustainability and gentleness to the environment.^6–8^

The overall efficiency of the selected photocatalysts can be adapted by modifications and surface functionalization.^9,10^ The synergy between the structure and decorations can be assessed by nanoarchitectonics that represents a modern tool in materials science for the fabrication of dynamic advanced materials.^11^ Nanoarchitectonics represents a concept that exploits the relationship among materials, molecular design, and overall functionality. It organizes functional materials from the atomic-level scale into macroscopically operating advanced dynamic systems of a high degree of organization.^12^ The tools of nanoarchitectonics are universal and can also be applied in the field of photocatalysis and energy conversion.^13^ In the latest research trends in this regard, the use of atomic-level species helps to increase the photocatalytic efficiency per unit mass while reducing the amount of required material.^14–16^ Especially, Pt in the form of single atoms or atomic-level species has shown great efficiency as a photocatalyst for the HER.^17–19^ Recently, the implementation of single atoms as dynamic catalysts has been reported in the field of nano/microrobotics.^20,21^ Atomic-level precise engineering was demonstrated to improve the control over propulsion capabilities and provide nano/microrobots with catalytic abilities for environmental remediation and medical applications.^22,23^

Nano/microrobots are autonomous, highly functional nano/microdevices that are physically and chemically programmed to accomplish a specific task.^24^ The main advantage lies in performing the accomplishment in a so-called “on-the-fly” mode that is given by their propulsion abilities.^25,26^ The propulsion abilities can be provided by chemically fueling the nano/microrobots by using a specific chemical reaction to generate nonhomogeneous gradients that lead to the propulsion.^27^ Alternatively, nano/microrobots can be propelled in external fields, such as light irradiation, magnetic or electric field, ultrasound, etc.^28,29^ Providing nano/microrobots with propulsion abilities, “on-the-fly” action, navigation to areas with limited access, or collection after finalizing the task can be achieved.^30^ Because of all abilities, a promising efficiency of nano/microrobots has been observed in the field of catalysis and photocatalysis. Nano/microrobots can catalyze chemical conversion, photocatalytically degrade pollutants, and convert analytes not only in medical applications but also in environmental remediation, food industry, etc.^31–35^ Lately, nano/microrobots have been demonstrated to even aid in energy conversion applications.^36^ In a recent publication, Mallick et al.^37^ reported on ammonia production from nitrates through photosynthesis using photocatalytic microrobots as “on-the-fly” photocatalysts.

Here, we fabricated photocatalytic microrobots derived from black titanium oxide bTiO_2_ and optimized their propulsion abilities toward the HER by decorating their surface with magnetic nanoparticles (NPs) (microrobots are referred to as mag-bTiO_2_ microrobots). To increase the overall photocatalytic efficiency in the HER, the_2_ microrobots were decorated with Pt atomic-level species to fabricate mag-Pt-bTiO_2_ microrobots. The schematic illustration of the nanoarchitectonics of microrobots is presented in Figure 1. The impact of different surface modifications on the photocatalytic HER was systematically evaluated, as well as the contribution of the dynamic character of the microrobots operating in the “on-the-fly” mode. The results clearly demonstrate the applicability of microrobots in energy conversion applications and pave the way toward a next-generation nano/microrobots.

**Figure 1:**
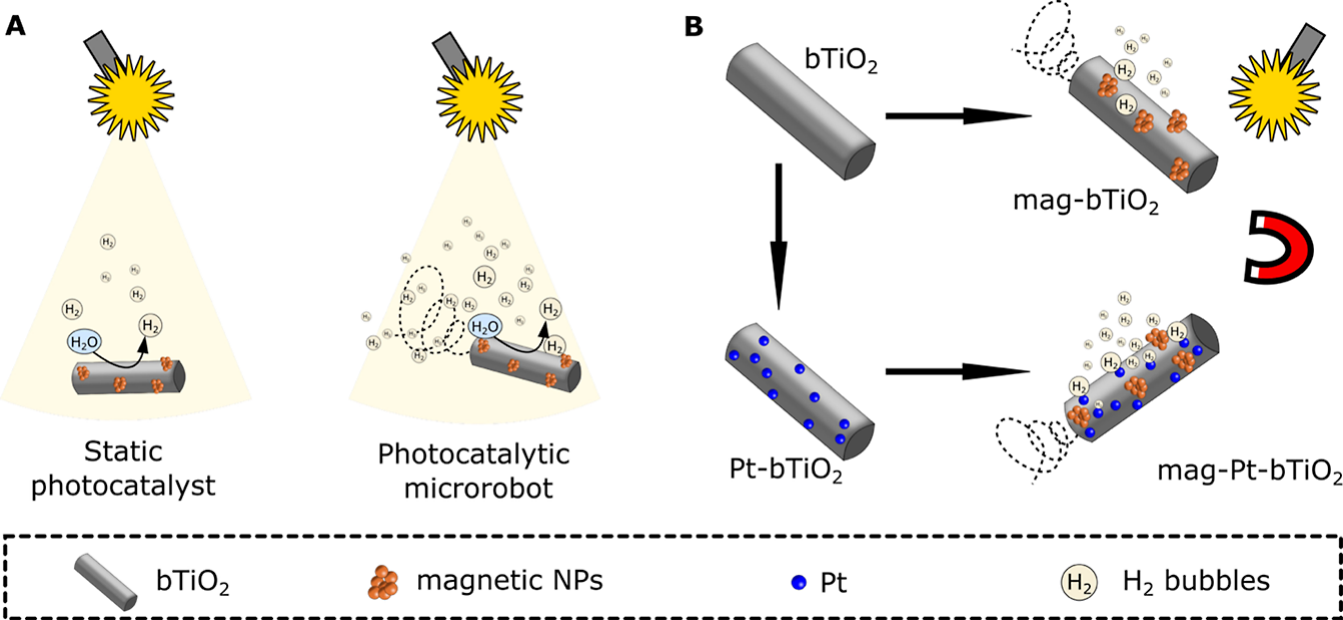
A schematic illustration representing the nanoarchitectonics of microrobots for the HER. (A) Comparison of the use of static photocatalysts and photocatalytic microrobots in the “on-the-fly” mode for the photocatalytic HER. (B) Approach to the fabrication of microrobots for the HER.

## Results and Discussion

The microrobots were derived from TiO_2_ anisotropically grown microstructures that were synthesized by a hydrothermal method reported by Ullattil and Pumera.^38^ As a result of the synthetic approach, rod-like microparticles of the anatase phase were obtained (Figures S1 and 2A,B). Although anatase already has significant photocatalytic properties, we subsequently performed surface reduction in a H_2_ atmosphere to implement surface defects to potentially increase photocatalytic efficiency by broadening the light absorption range.^39^ The resulting defect-rich TiO_2_ microrobots, referred to as bTiO_2_ microrobots, were obtained. The absorption spectrum (Figure 2A) of the bTiO_2_ microrobots shows the shift in the onset of absorption from 394 to 520 nm after the reduction procedure in the H_2_ atmosphere. The shift suggests a broadening of the band gap, which implies that a wider light spectrum can be employed for photocatalytic processes.^40^ It is worth noting that no significant changes in the crystalline structure were observed after the reduction process; the diffractogram of the bTiO_2_ microrobots corresponded to the diffractogram of a model anatase phase (Figure 2B).

**Figure 2:**
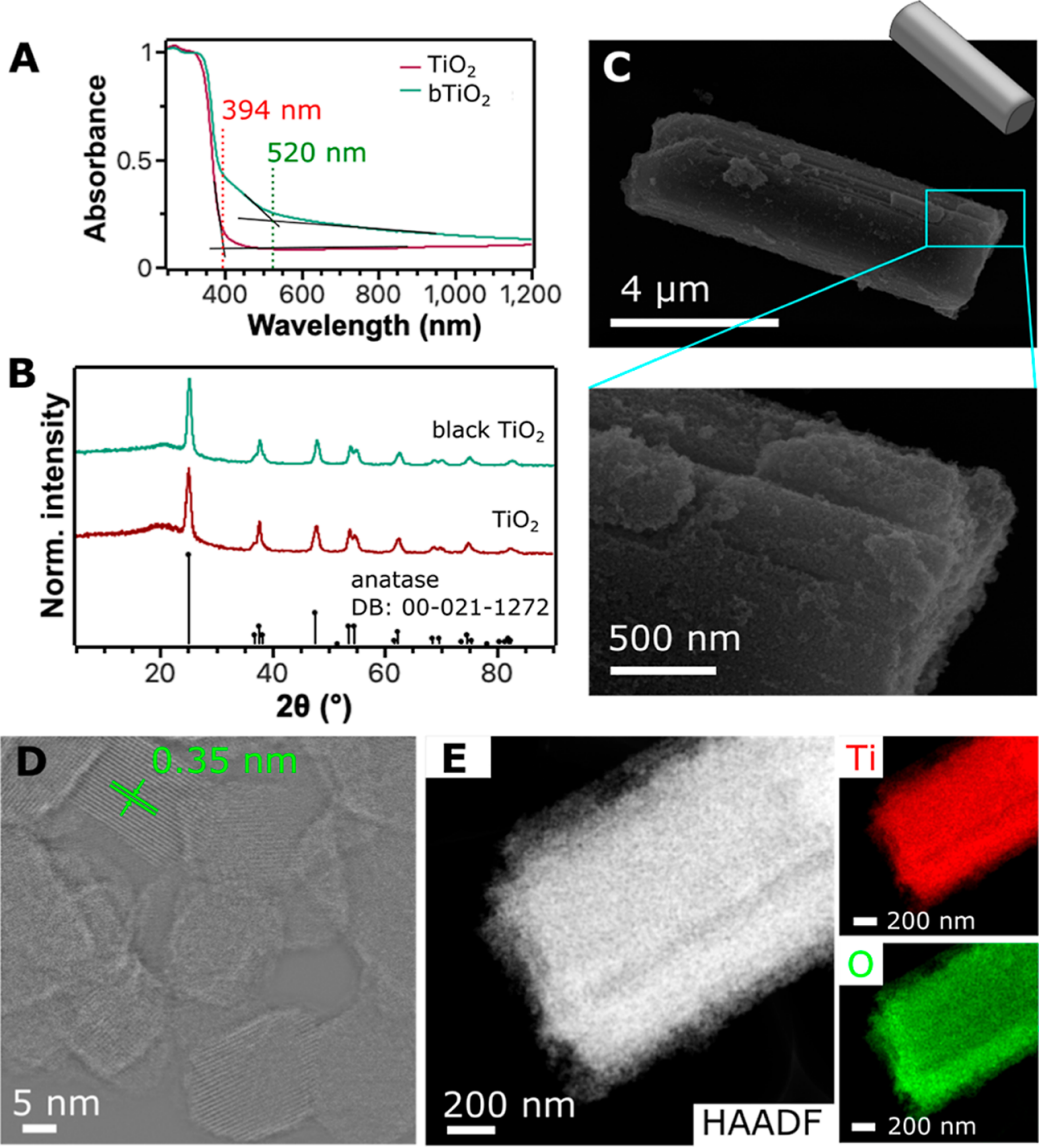
Optical and structural characterization of bTiO_2_ microrods. (A) Absorption spectra of as-prepared TiO_2_ and bTiO_2_ materials with the highlighted onset of absorption. (B) Powder XRD diffractograms of TiO_2_ and bTiO_2_ compared to the anatase diffractogram. (C) SEM micrograph of a representative bTiO_2_ microrod with a detailed view of the surface morphology. (D) HAADF-STEM characterization of a bTiO_2_ microrod with a labeled interlayer spacing. The original micrograph was processed using a high-pass filter. (E) Elemental mapping of the bTiO_2_ microrod that shows the presence of oxygen and titanium.

The structural characterization of the bTiO_2_ microrobots revealed a rod-like morphology (Figure 2C) that was observed already in the as-synthesized anatase TiO_2_ microparticles (Figure S1). As the micrograph from scanning electron microscopy (SEM) characterization shows in detail (Figure 2C), the mesocrystal morphology can be observed. Closer structural characterization using high-angle annular dark field scanning transmission electron microscopy (HAADF-STEM) (Figures 2D, S2, and S3) revealed the mesocrystal morphology in which the resulting microrods were formed by nanosized crystals of a highly organized crystalline structure. The lattice spacing was 0.35 nm, which corresponds to the (101) lattice plane of anatase.^41^ Energy-dispersive X-ray (EDX) spectroscopy characterization demonstrated the homogeneous presence of Ti and O within the structures of microrobots (Figures 2E and S4).

Before considering bTiO_2_ microparticles for the application as microrobots in energy conversion, their photocatalytic properties toward the HER were evaluated (Figure S5). The HER was carried out in a 10 vol % methanol solution, where methanol acted as a sacrificial reagent,^42^ under the exposure to a Xe lamp light source in a gas-tight system. The reaction mixture containing bTiO_2_ microparticles was magnetically stirred using a magnetic bar, and H_2_ generation was monitored by a gas chromatograph coupled with a thermal conductivity detector (GC-TCD) for 5 h. As a result, the total H_2_ yield was 290 μmol after 5 h with a reaction rate that reached 60 μmol/h. Confirming the photocatalytic ability to generate H_2_, bTiO_2_ microparticles were evaluated as suitable starting blocks for nanoarchitecting microrobots for the HER.

In the nanoarchitectonics of microrobots, providing propulsion abilities is the most crucial step. Propulsion abilities differentiate nano/microrobots from their static counterparts by enabling performance in a “on-the-fly” mode that improves efficiency in a task accomplishment, such as catalysis, degradation, capture, signaling, etc.^21,43^ Therefore, the propulsion abilities of the bTiO_2_ microparticles were tested in the next step to evaluate their applicability as microrobots. Previously published work demonstrated that nano/microrobots derived from bTiO_2_ exhibit photocatalytic abilities that allow their propulsion under light irradiation and eventually in the presence of hydrogen peroxide (H_2_O_2_) that acts as a chemical fuel.^44^

Taking this behavior into account, we tested the propulsion abilities of bTiO_2_ microrobots under light irradiation in the presence of H_2_O_2_. In particular, microrobots were observed in their colloidal solutions of 1 wt % H_2_O_2_ under light illumination of different wavelengths ranging from 360 to 650 nm (Figure 3A–E). To distinguish the difference between dark and illuminated conditions, the microrobots were kept in dark conditions for 10 s and then exposed to irradiation for 20 s. Subsequently, the light source was switched off, and the colloidal solution was observed for another 30 s. The results summarized in Figure 3 clearly demonstrate schooling behavior, i.e., clustering of microrobots into aggregates and their reversible expansion under light irradiation. Light-induced schooling behavior of TiO_2_-based microrobots has been reported in previous works.^38^ The clustering originates from electrolyte diffusiophoresis generated among TiO_2_ microrobots that spontaneously generate a local electric field in their immediate environment by undergoing acid–base reactions over acidic surface OH groups. The presence of H_2_O_2_ as a fuel supports acid–base reactions, and thus, clustering is supported to a greater extent. Upon light irradiation, H_2_O_2_ is photocatalytically decomposed over bTiO_2_ microrobots, and the generated chemical gradients induce a chemiosmotic slip that causes expansion of the microrobot clusters. In dark conditions, the photocatalytic decomposition of fuel does not occur anymore, and therefore, the acid–base reactions leading to the clustering effects dominate.^45,46^ As expected, there was a significant difference in the degree of schooling behavior when different light sources were used (Figure 3). UV light (360–370 nm) and violet light (383–408 nm) supported the expansion abilities of the clusters to individual microrobots. The expansion efficiency decreased with increasing wavelength. This observation is in correlation with the findings from optical spectroscopy characterization (Figure 2A). Light absorption was the most effective below 395 nm, meaning that the most efficient photocatalytic abilities are induced under light irradiation within the UV region. However, a certain degree of the cluster expansion was observed even when applying an orange LED source (550–590 nm), suggesting efficient light absorption in the visible range, which is consistent with the optical properties of black titanium oxide materials.^44^

**Figure 3:**
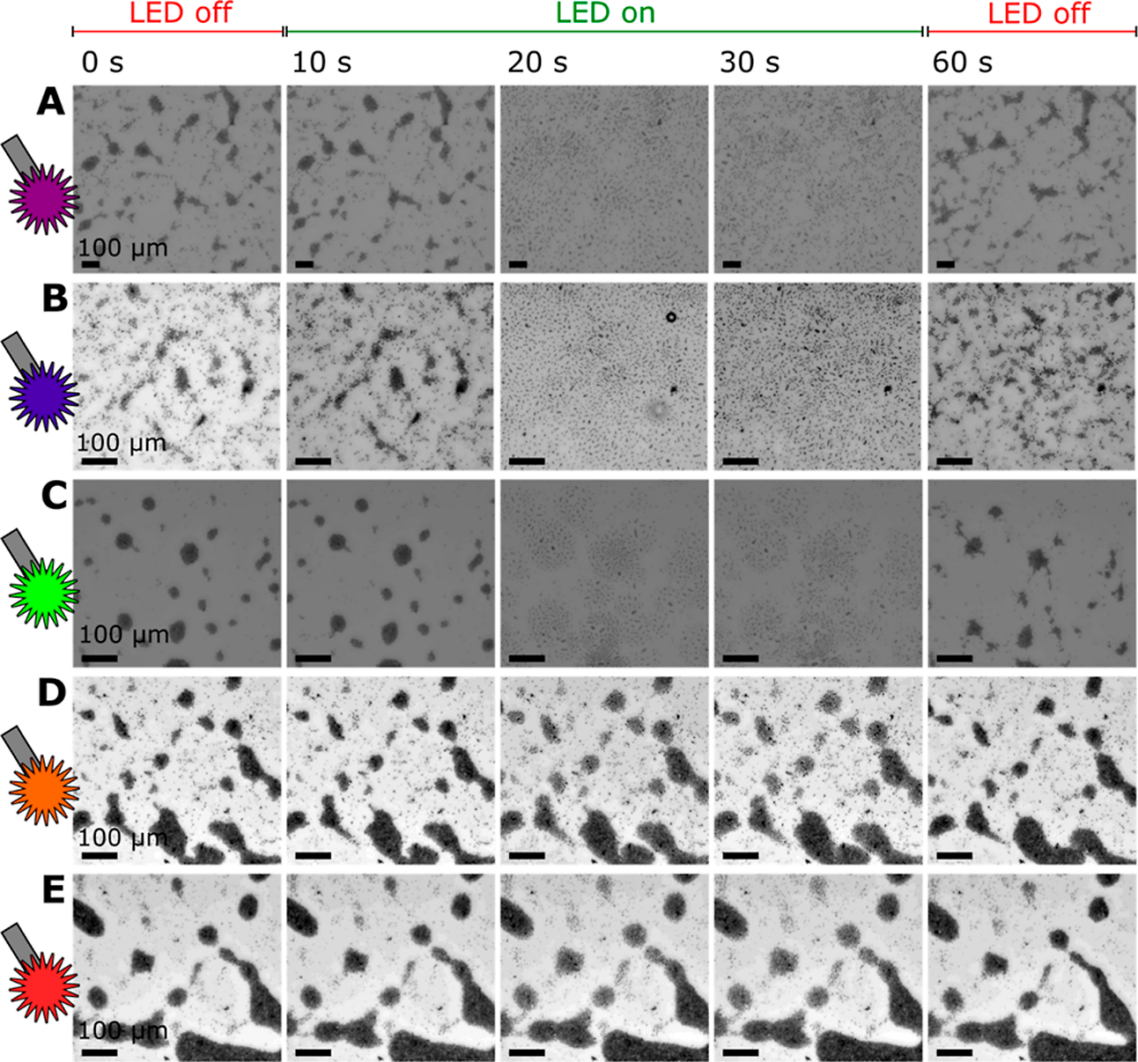
Light-induced propulsion and schooling behavior of bTiO_2_ microrobots in the presence of 1 wt % H_2_O_2_. The microrobots were observed for 60 s (0–10 s under dark conditions, 10–30 s under light irradiation, and 30–60 s under dark conditions). LED sources provided wavelengths of (A) 360–370 nm, (B) 383–408 nm, (C) 460–500 nm, (D) 550–590 nm, and (E) 590–650 nm. The scale bar is 100 μm for all micrographs.

Traditional approaches of nanoarchitectonics in the field of nano/microrobotics aim to propel the nano/microrobots in multiple modes.^47^ In photocatalytic applications, fuel- and/or light-induced propulsion is often designed in conjunction with magnetic abilities.^48^ While fuel- and/or light-induced propulsion supports “on-the-fly” action, magnetic field-driven nano/microrobots can be efficiently navigated, concentrated in a desired location, and collected to enable regeneration processes and potential reusability.^49,50^ Moreover, the propulsion capabilities induced by the magnetic field are independent of the environments of different ionic strengths and solvent mixtures.^51^ Taking into account the advantages of multimodal propulsion abilities, we decorated the microrobots with magnetic nanoparticles to fabricate magnetic bTiO_2_ microrobots, referred to as mag-bTiO_2_ microrobots, as illustrated in Figure 1. The magnetic nanoparticles of Fe_2_O_3_ described in previous publications were used.^51,52^ The decoration was carried out by incubating the bTiO_2_ microrobots in a 50 vol % ethanol solution containing 10 wt % magnetic NPs with respect to the amount of bTiO_2_ microrobots following a procedure reported in a previous work.^51^ Figure 4A shows an SEM micrograph of a mag-bTiO_2_ microrobot. Successful decoration of the microrobots with magnetic NPs was demonstrated by performing EDX elemental mapping. Figure S6 confirms the nonhomogeneous distribution of the iron signal in the form of clusters over the body of a representative mag-bTiO_2_ microrobot. The resulting magnetic field-driven propulsion abilities were verified by exposing mag-bTiO_2_ microrobots to a magnetic field induced by a permanent neodymium magnet. The corresponding micrographs that capture the magnetic navigation are presented in Figure 4B.

**Figure 4:**
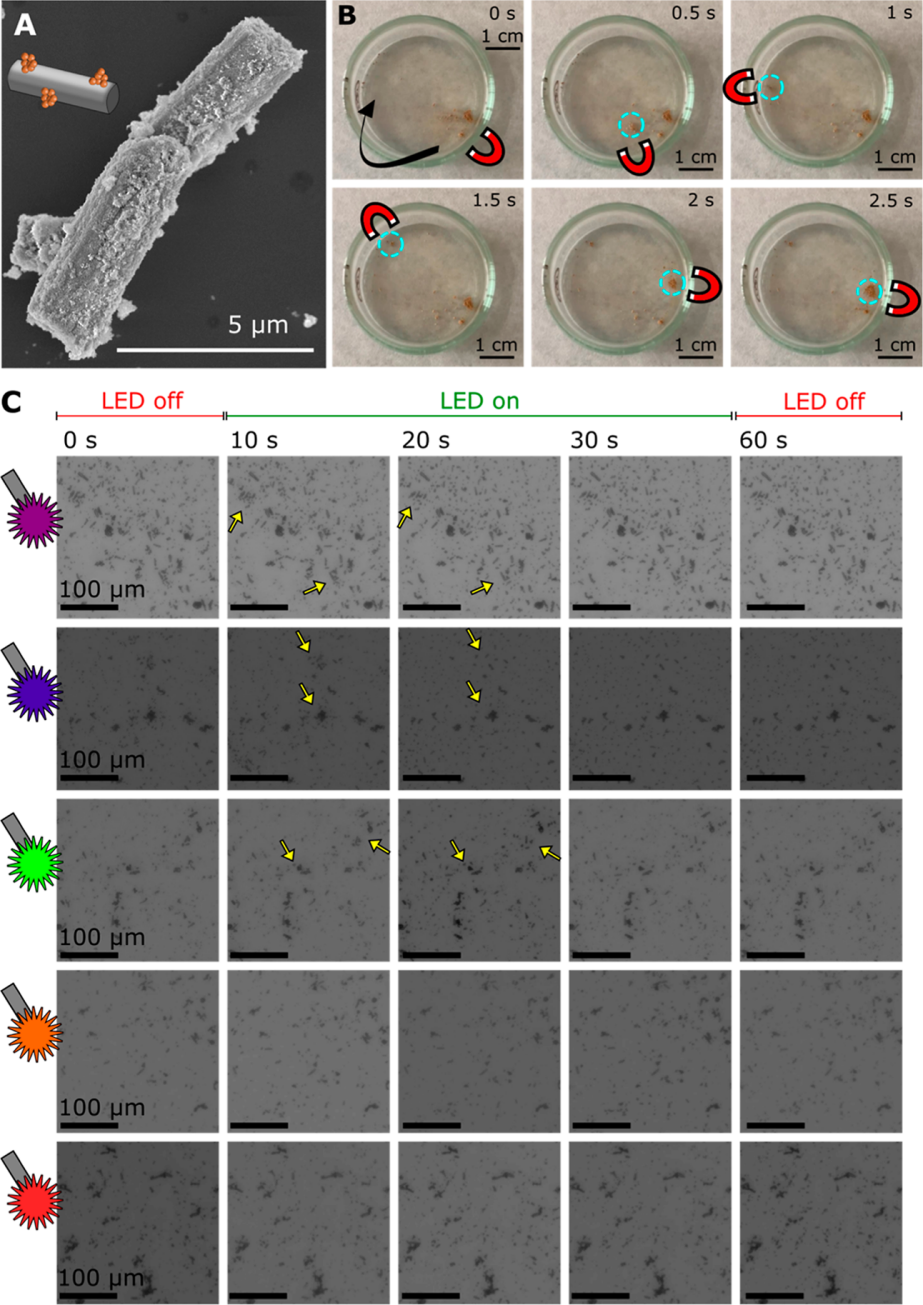
Fabrication of magnetic microrobots and their propulsion abilities. (A) A SEM micrograph of mag-bTiO_2_ microrobots. (B) Navigation of magnetic microrobots in an external magnetic field induced by a permanent neodymium magnet that was operated below the Petri dish. The actual position of the permanent magnet is indicated by its icon in the corresponding photographs. (C) Light-induced propulsion of microrobots in 10 vol % methanol using light irradiation of different energies. The microrobots were observed for 60 s (0–10 s under dark conditions, 10–30 s under light irradiation, and 30–60 s under dark conditions). The LED sources provided wavelengths of 360–370 nm, 383–408 nm, 460–500 nm, 550–590 nm, and 590–650 nm. The scale bar is 100 μm for all micrographs.

To evaluate the influence of surface modifications on light-induced propulsion abilities, mag-bTiO_2_ microrobots were tracked under light irradiation. This time, the conditions of the reaction mixture were simulated to evaluate the propulsion abilities during HER. Following the HER procedure, the colloidal solution was prepared in a 10 vol % methanol solution, and no fuel was added to increase the propulsion of the microrobots. It should be noted that the use of methanol as a sacrificial agent in the HER is crucial to support the charge separation by removing the generated holes, thus leaving the electrons available to produce hydrogen.^55^ As a result, the modifications of the material and the environment significantly suppressed schooling behavior. Figure 4C captures schooling behavior when irradiating mag-bTiO_2_ microrobots with UV (360–370 nm), violet (383–408 nm), and green (460–500 nm) LEDs. Although schooling behavior was not as significant as in the tracking experiments with bTiO_2_ microrobots (Figure 3), the propulsion abilities were still observed. Interestingly, the expansion of the clusters of the microrobots under light irradiation was not reversible this time; i.e., no contraction of the clusters was observed when light irradiation was turned off. This phenomenon is attributed to the presence of methanol that acts as a sacrificial agent in the HER. In particular, methanol captures the generated holes that are responsible for the generation of electrical and chemical gradients, which induce the phoretic forces.^56^ This fact could also explain the observation that no schooling behavior was observed when applying light of wavelengths above 550 nm. As demonstrated in Figure 2A, the onset of absorption of the bTiO_2_ material occurs around 520 nm; at this edge, the light absorption and charge generation might not be sufficient to induce propulsion of microrobots, especially in the absence of chemical fuel and in the presence of methanol that captures the generated holes carrying a positive charge. Considering the overall findings, the mag-bTiO_2_ microrobots were applied for the HER in the following steps while using both light and magnetic field-induced propulsion abilities to achieve H_2_ photocatalytic generation in the “on-the-fly” mode.

Photocatalytic HER experiments were carried out by dispersing mag-bTiO_2_ microrobots in a 10 vol % methanol solution and exposing the reaction mixture to light irradiation, following the procedure of the preliminary experiment with bTiO_2_ microparticles. To assess the efficiency of the photocatalytic “on-the-fly” mode, the mag-bTiO_2_ microrobots were propelled in dual modes. First, the Xe light source was applied not only as a source for the photocatalytic generation of hydrogen but also as a source to propel the microrobots (Figure 5A). Furthermore, the reaction mixture was placed on a magnetic stirrer to be stirred externally at 500 rpm by using the propulsion abilities of microrobots without using any magnetic bar. The photographs in Figure 5B show the reaction mixture before and after completing the HER after 5 h. Clearly, a certain degree of sedimentation occurred, indicating a nonhomogeneous distribution of microrobots as photocatalysts in the reaction mixture during the reaction. However, a vortex pattern is observed at the bottom of the reaction container, demonstrating that the magnetic propulsion of mag-bTiO_2_ microrobots had been efficient during the reaction. As a result, the total H_2_ yield was 1.3 ± 0.7 μmol in 5 h (Figure 5C); the H_2_ production rate increased in 4 h and then reached its maximum of 0.36 μmol/h (Figure 5D). Interestingly, the extended time led to a drop in the H_2_ production rate. The morphology of mag-bTiO_2_ microrobots was assessed using SEM and EDX techniques after completing the HER to evaluate possible surface changes and eventually washing out magnetic nanoparticles that could contribute to the elimination of propulsion abilities, explaining the drop in the efficiency. However, the structural characterization did not reveal any significant changes in the morphology or elemental composition of mag-bTiO_2_ microrobots (Figure S7). The reason for the drop in the H_2_ production rate could originate from the formation of byproducts or other changes in the reaction mixture or oversaturation of the reaction sites and H_2_ bubble accumulation on the surface of the photocatalyst.^53,54^ To evaluate the contribution of the “on-the-fly” mode to the overall photocatalytic efficiency in the next step, a control experiment without any external magnetic stirring was conducted. In the control experiment, the magnetic stirrer was left under the reaction mixture to provide permanent magnetic forces that enhanced the sedimentation of the magnetic microrobots and supported their static character. In static conditions, the total H_2_ yield was 0.45 ± 0.07 μmol in 5 h, suggesting a 65% drop in the yield compared to the efficiency of microrobots in the propelled mode (Figure 5C). The profile of the reaction rate with time was comparable; the maximum reaction rate of 0.14 μmol/h was observed after 4 h, and then a drop was observed (Figure 5D). These results suggest the successful photocatalytic production of H_2_ using microrobots and a positive contribution of their “on-the-fly” regime.

**Figure 5:**
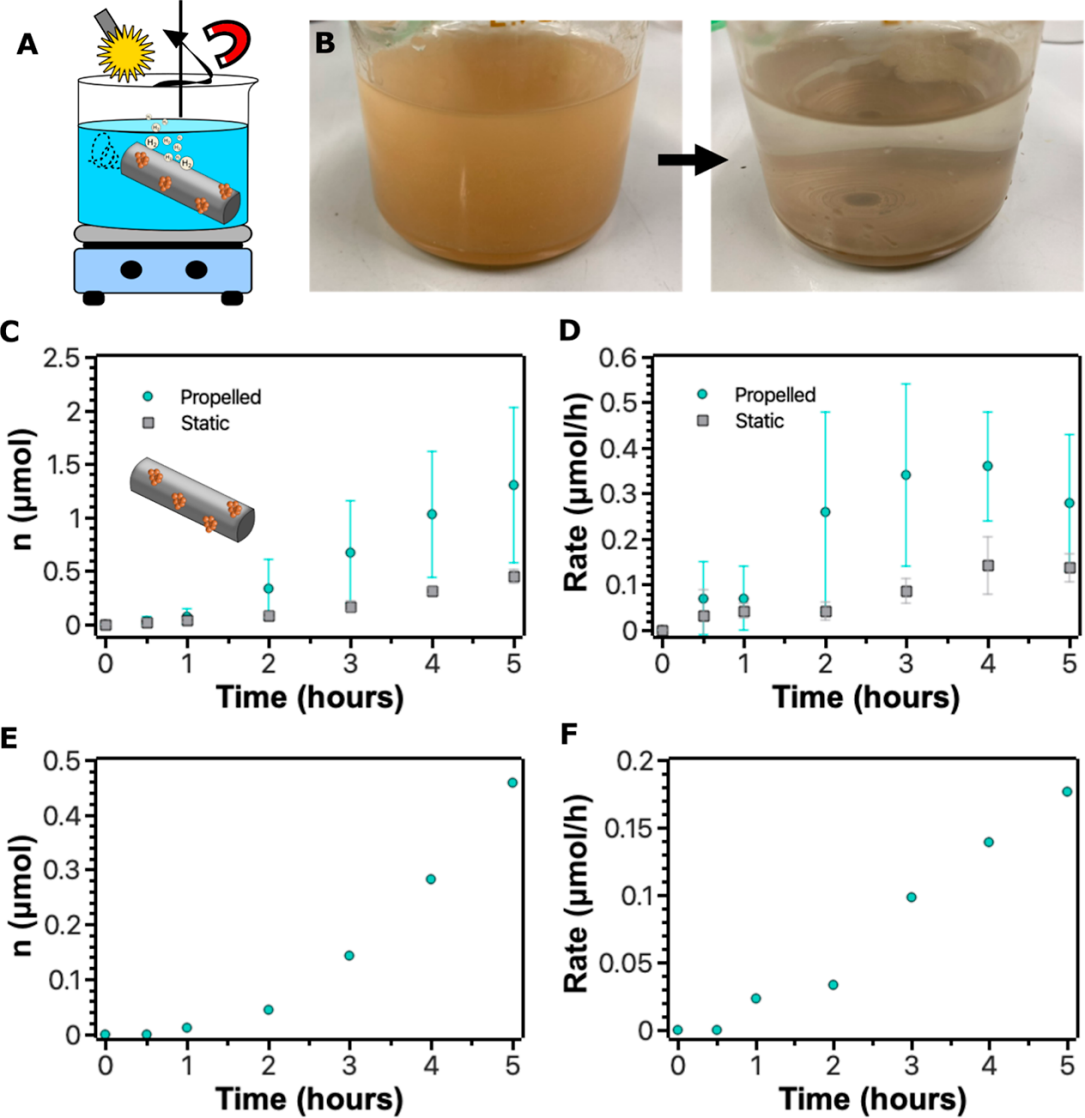
Mag-bTiO_2_ microrobots as “on-the-fly” photocatalysts for the HER. (A) Illustration of the HER using mag-bTiO_2_ microrobots. (B) Photographs of the reaction mixture before and after the HER. (C,D) H_2_ yields and the reaction rate of the HER, respectively, over time by using mag-bTiO_2_ microrobots as photocatalysts and a comparison of propelled and static systems. The values and the standard deviation were determined from 3 independent experiments. (E,F) Recyclability experiments demonstrating yields of H_2_ and the reaction rate of the HER, respectively, over time by using recovered mag-bTiO_2_ microrobots as photocatalysts in the “on-the-fly” mode.

In general, the “on-the-fly” regime supports the dynamics in the reaction mixture by generating its flow in the immediate environment of the microrobots that act as photocatalysts. From this point of view, it could be assumed that supporting the dynamic character of microrobots by overcoming the inhibition of the light-induced propulsion of the microrobots in the reaction mixture could even enhance the efficiency of the HER. The application of light irradiation pulses might even improve the efficiency of the HER in future settings as it could eventually support reversible cluster formation and expansion that would induce a flow of the reaction mixture.

Considering the possible reusability of the microrobots as “on-the-fly” photocatalysts for the HER, they were collected after the reaction was completed and reused without any additional regeneration steps. As presented in Figure 5E and F, the total yield of H_2_ was 0.46 μmol in 5 h, which was lower compared to the efficiency of freshly prepared microrobots. The maximum reaction rate was 0.18 μmol/h after 5 h of reaction. The decrease in the efficiency is most likely caused by a lower concentration of the photocatalyst that was collected magnetically using an external permanent magnet. This method of collection was straightforward as it exploited the propulsion abilities of the microrobots; however, it might not have been a fully quantitative approach.

One of the current trends in the field of photocatalysis is the employment of catalysts in the form of single atoms and atomic-level species. This approach of nanoarchitectonics increases the efficiency of catalytic processes by utilizing active sites with great control and by reducing the amount of costly metallic catalysts.^57^ This trend has also been applied in the field of photocatalytic HER processes by employing Pt single atoms as photocatalysts.^58,59^ Motivated by the great efficiency of single Pt atoms as photocatalysts, we decorated bTiO_2_ microrobots with single-atomic-level Pt species via a solution impregnation technique (Figure 1). The resulting Pt-bTiO_2_ microrobots had a rod-like shape with a mesocrystal morphology as in the case of bTiO_2_ microrobots (Figure 6 A–C). HAADF-STEM characterization also confirmed the presence of atomic-level Pt species on the surface of the Pt-bTiO_2_ microrobots. It is worth noting that Pt was dispersed in the form of single atoms and atomic clusters homogeneously, as demonstrated by an elemental mapping; the corresponding EDX spectra are provided in Figure S8. To further support the statement that Pt was present in the form of atomic-level species, XPS characterization was performed (Figures 6D and S9). In addition to Ti and O, a signal of Pt was detected in the high-resolution (HR) spectra. Peaks of Pt 4*f*_5/2_ and Pt 4*f*_7/2_ exhibited their maxima at 76.3 and 73.1 eV, respectively, suggesting the existence of Pt in the form of Pt^δ+^, where 0<δ < 2.^22^

**Figure 6:**
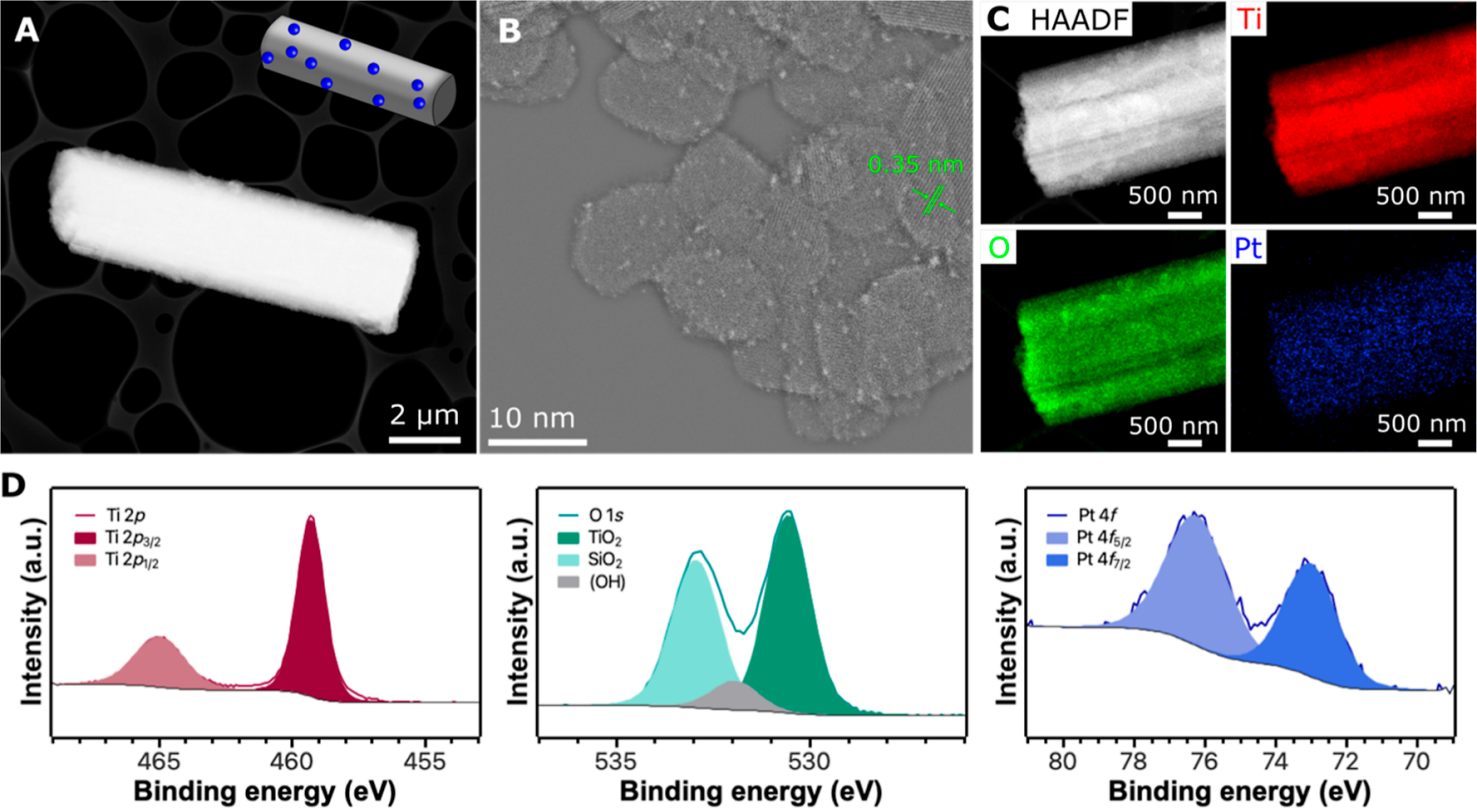
Structural characterization of Pt-bTiO_2_ microrobots. (A) A HAADF-STEM micrograph of a representative Pt-bTiO_2_ microrobot. (B) High-resolution micrograph of the Pt-bTiO_2_ microrobot demonstrating the presence of atomic-level Pt species distributed over the surface. The micrograph was processed by applying a high-pass filter. (C) Elemental mapping of a Pt-bTiO_2_ microrobot revealing the presence of Ti, O, and Pt. (D) XPS characterization and HR spectra of Ti 2*p*, O 1*s*, and Pt 4*f*.

To verify the photocatalytic efficiency of the Pt-bTiO_2_ microparticles without considering them as microrobots, the HER was performed in the same setting as when applying bTiO_2_ microparticles as photocatalysts (Figure S5). Briefly, Pt-bTiO_2_ microparticles were dispersed in a 10 vol % methanol solution and exposed to a Xe light source while being magnetically stirred using a magnetic bar. The photocatalytic efficiency increased significantly compared to the bTiO_2_ microparticles: the total yield of H_2_ was 1890 μmol in 5 h (Figure S10A), which is 6.5 times higher than when the unmodified bTiO_2_ microparticles were used as photocatalysts (Figure S5). Surprisingly, the rate of H_2_ generation tended to decrease with time (Figure S10B); the rate of 450 μmol/h decreased to the rate of 310 μmol/h from 1 to 5 h of reaction time. To explain this observation, the photocatalyst was collected after completing the reaction and characterized using HAADF-STEM and XPS analysis. Figure S11 shows HAADF-STEM micrographs of Pt-bTiO_2_ microparticles with formed Pt clusters. Clearly, atomic-level Pt species aggregated into nanosized clusters during the HER. The dynamic character of single Pt atoms and atomic-level species, especially under harsh conditions, has already been discussed in previously published works.^60,61^ The formation of Pt clusters was also assessed by XPS characterization (Figure S12). The HR Pt 4*f*_7/2_ peak was deconvoluted into two peaks centered at 71.5 and 72.4 eV, pointing out the coexistence of metallic Pt^0^ and Pt^δ+^ species, respectively.^22^ The photocatalytic HER catalyzed by metallic Pt in the form of nanoparticles has already been reported to have a lower efficiency when compared to the reaction catalyzed by atomic-level Pt species.^62^ Therefore, it was concluded that the significant drop in the reaction rate (Figure S10B) most likely originated from the aggregation of Pt atomic-level species.

As a result, the HER efficiency increased when using the Pt-bTiO_2_ material instead of bTiO_2_ microparticles in the traditional setting of the photocatalytic HER when a magnetic stirring bar was used. Motivated by the improved efficiency, we transformed Pt-bTiO_2_ microparticles into mag-Pt-bTiO_2_ microrobots by a consecutive decoration with magnetic NPs and compared the photocatalytic HER efficiency with that of their mag-bTiO_2_ counterparts (Figure 1). Initially, the successful attachment of magnetic NPs was verified by performing SEM and EDX analysis (Figure S13). In the next step, propulsion abilities were studied since the presence of atomic-level species can significantly affect the resulting propulsion abilities of microrobots.^22,63^ Light-induced propulsion abilities were tested in a simulated reaction mixture to reveal any possible effect of surface modifications prior to the use of mag-Pt-bTiO_2_ microrobots as “on-the-fly” photocatalysts. Briefly, mag-Pt-bTiO_2_ microrobots were dispersed in a 10 vol % methanol solution without the use of a chemical fuel and exposed to light irradiation of different wavelengths (Figure 7). In agreement with previous experiments with mag-bTiO_2_ microrobots (Figure 4C), the schooling behavior of microrobots was suppressed under light irradiation; i.e., the expanded clusters did not cover a significant area as in the case of fueled bTiO_2_ microrobots studied in a fully aqueous environment (Figure 3). However, the propulsion abilities were still observed up to a certain point. In the case of mag-Pt-bTiO_2_ microrobots, schooling behavior was induced by using the light sources up to 590 nm (Figure 7D). The extended wavelength range for the propulsion of Pt-bTiO_2_ microrobots when compared with their bTiO_2_ counterparts could originate from the synergistic contributions of the bTiO_2_ material and Pt decoration. Black titanium oxide can absorb visible light due to the presence of oxygen vacancies and defect states, while Pt nanoparticles can enhance photocatalytic activity either via electron trapping or through plasmonic effects.^64–67^ Finally, it should be noted that the expansion ofmicrorobots under light irradiation was irreversible.

**Figure 7:**
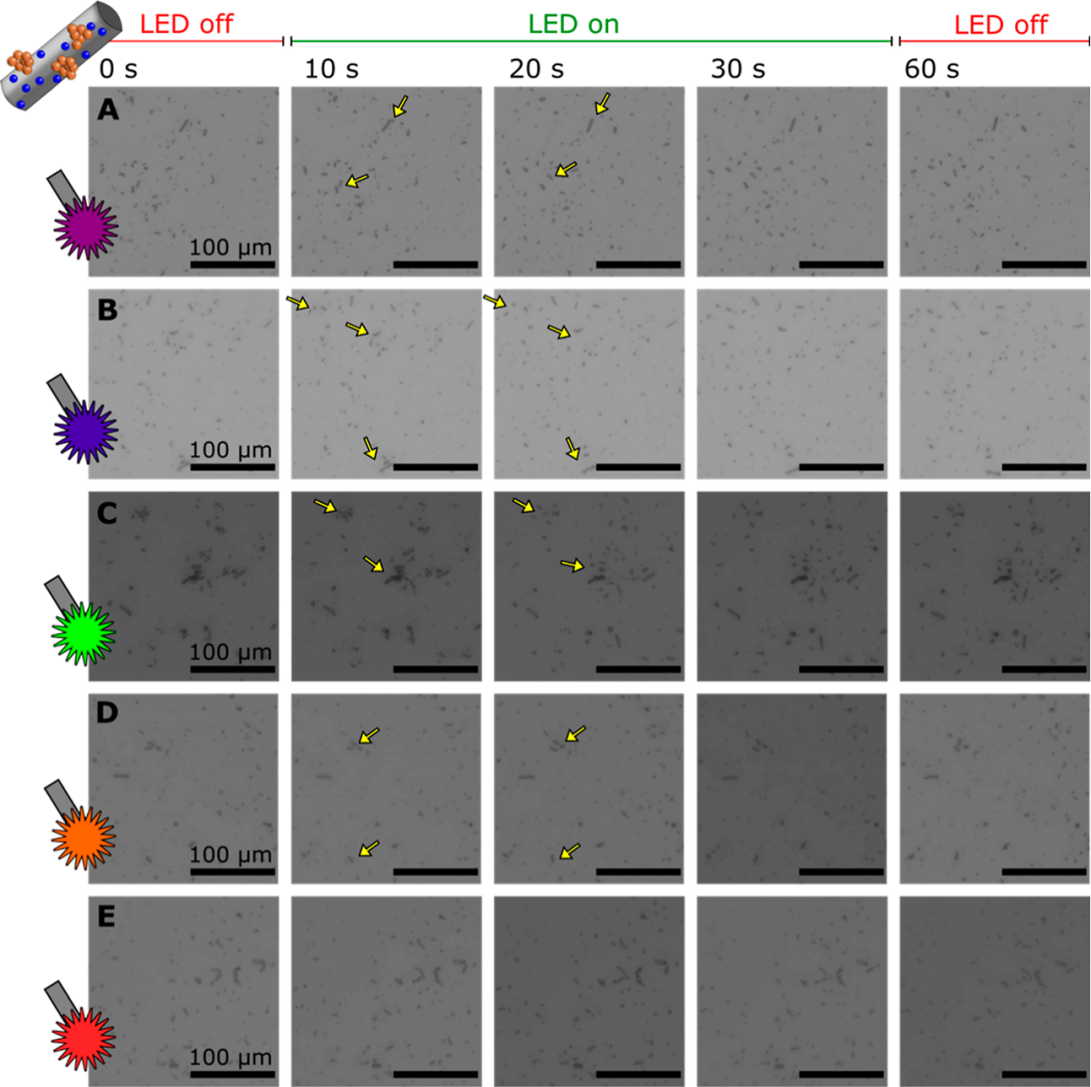
Light-induced propulsion of mag-Pt-bTiO_2_ microrobots in 10 vol % methanol. The microrobots were observed for 60 s (0–10 s under dark conditions, 10–30 s under light irradiation, and 30–60 s under dark conditions). LED sources provided wavelengths of (A) 360–370 nm, (B) 383–408 nm, (C) 460–500 nm, (D) 550–590 nm, and (E) 590–650 nm. The scale bar is 100 μm for all micrographs.

Finally, mag-Pt-bTiO_2_ microrobots were tested as “on-the-fly” photocatalysts for the HER following the procedure as when using mag-bTiO_2_ microrobots. As a result, the total yield of H_2_ was 13 ± 7 μmol in 5 h when mag-Pt-bTiO_2_ microrobots were applied as “on-the-fly” photocatalysts (Figure 8). It is worth noting that the modification with atomic-level Pt species increased the HER efficiency about 10 times when compared with the reaction performed by mag-bTiO_2_ microrobots (Figure 5C). Interestingly, the reaction rate profile (Figure 8D) was different from that when mag-bTiO_2_ microrobots were applied. While the use of mag-bTiO_2_ microrobots caused an increase in the reaction rate until it reached a plateau after 2–3 h, the use of mag-Pt-bTiO_2_ microrobots showed a decreasing trend in reaction rate over time. The maximum reaction rate for H_2_ generation was observed at 0.5 h, reaching 8 ± 2 μmol/h, and then it dropped to 2 μmol/h after 2 h. These results correspond to the preliminary observation of the efficiency of the Pt-bTiO_2_ microparticles (Figure S10). In that case, aggregation of atomic-level Pt species occurred, and it is reasonable to assume the same kind of degradation in the case of using mag-Pt-bTiO_2_ microrobots as photocatalysts. It is worth noting that no additional changes in the morphology were observed as verified by SEM and EDX analysis (Figure S14).

**Figure 8:**
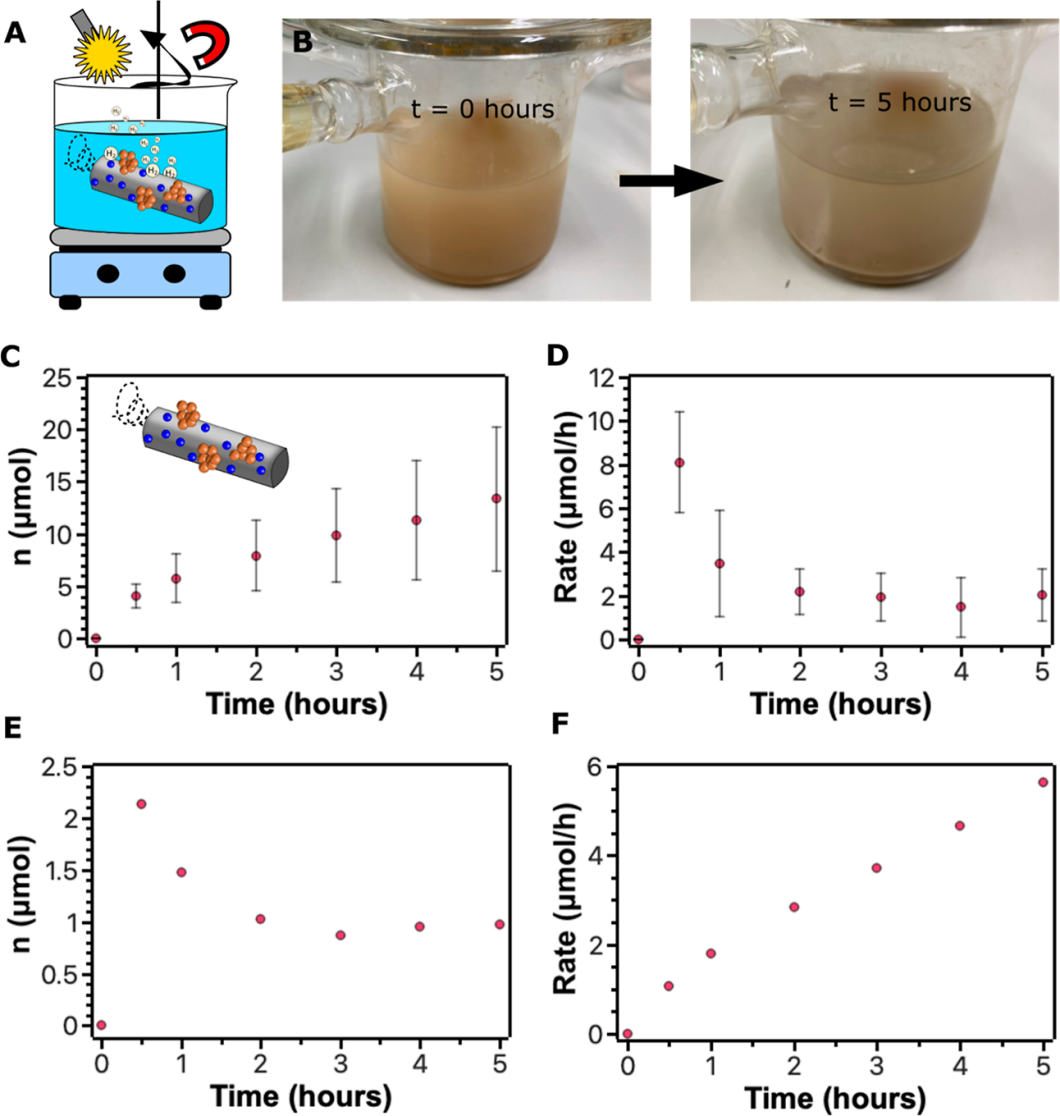
(A) Experimental setup of the HER using mag-Pt-bTiO_2_ microrobots as photocatalysts. (B) Photographs of the reaction mixture before and after the HER. (C,D) Yields of H_2_ and the reaction rate of the HER, respectively, over time using mag-Pt-bTiO_2_ microrobots as photocatalysts. The values and the standard deviation were determined from 3 independent experiments. (E,F) Recyclability experiments demonstrating H_2_ yields and the reaction rate of the HER, respectively, over time using recovered mag-Pt-bTiO_2_ microrobots as photocatalysts in the “on-the-fly” mode.

In addition, the possible reusability of mag-Pt-bTiO_2_ microrobots was studied to evaluate the sustainability of the targeted photocatalyst. Figure 8E,F demonstrates that the HER efficiency dropped significantly compared to the use of freshly prepared mag-Pt-bTiO_2_ microrobots. It is worth pointing out that the reaction rate profile was similar to that of the previous observations, which means that the reaction rate dropped over time, starting from 2.1 μmol/h at 0.5 h and stagnating around 1 μmol/h after 2 h. However, the decoration with Pt caused a significant increase in the efficiency of the HER, and the total yield of H_2_ was still higher when reusing the mag-Pt-bTiO_2_ photocatalysts than when applying unmodified mag-bTiO_2_ microrobots.

Comparing the overall results with previously published achievements, the application of microrobots toward the photocatalytic HER does not exceed the conventional TiO_2_ photocatalysts in our design. To give an example, Hejazi et al.^62^ decorated TiO_2_ anatase powder with Pt single atoms and tested their photocatalytic activity for the HER. Depending on the concentration of single atoms, the HER reached yields in the range of 1 to 5 mmol/h·g. In our work, the mag-Pt-bTiO_2_ microrobots reached a yield of up to 0.2 mmol/h·g in the HER, which is 1 order of magnitude lower in comparison with the aforementioned study. However, it is worth noting that the main advantage of using microrobots lies in their autonomous propulsion abilities and “on-the-fly” operation mode as well as wireless navigation and the possibility of retrieving and reusing the dynamic photocatalysts in subsequent processes. It is also worth noting that no systematic optimization of Pt loading was performed in this work. It can be expected that by more in-depth nanoarchitectonics of Pt decoration and by controlling the formation of single atoms, atomic-level species, and metallic nanoparticles, the efficiency could be further increased. Previous works have demonstrated that the concentration of Pt and the Pt^2+^/Pt^0^ ratio significantly influence the efficiency of the HER.^62,64,68^

## Conclusion

In conclusion, we designed magnetically navigated bTiO_2_-based microrobots as photocatalysts for the HER by following the principles of nanoarchitectonics. First, the propulsion abilities of mag-bTiO_2_ microrobots were evaluated in the magnetic field and under light irradiation to support the HER in the “on-the-fly” operation mode. The application of mag-bTiO_2_ microrobots as dynamic photocatalysts showed enhanced efficiency in H_2_ generation compared to that of their static counterparts. Moreover, the results demonstrate enhanced navigation in the magnetic field that enabled separation of microrobots from the reaction mixture and subsequent reusability, suggesting a potentially sustainable solution for microrobotics-assisted energy conversion. Further optimization of the microrobots by their decoration with atomic-level Pt species resulted in an increase of the yield of the HER by about 10 times. By following advanced principles of nanoarchitectonics and employing the latest findings in nanotechnologies to the field of nano/microrobotics, this proof-of-concept work presents a new avenue in the next-generation sustainable solutions in energy conversion applications beyond the photocatalytic HER.

## Experimental Part

### Fabrication of Microrobots

TiO_2_ microparticles were prepared by the following procedure. An aqueous solution of 0.1 M hexamethylenetetramine (HMTA) and 0.1 M titanium­(IV) oxysulfate (TiOSO_4_) was magnetically stirred for 1 h at room temperature. This was followed by the addition of 0.8 wt % NaOH in the volume ratio of 2:1 (titanium precursor solution to NaOH). The suspension was magnetically stirred for 1 h under ambient conditions and then transferred to an autoclave to perform a hydrothermal reaction at 150 °C for 18 h. The solid material was collected, washed with ethanol and distilled water three times, and dried at 60 °C overnight.

To introduce surface defects in the TiO_2_ structure, the TiO_2_ microparticles were reduced for 2 h at 500 °C in a hydrogen atmosphere using a T-furnace (heating rate: 5 °C/min). The resulting bTiO_2_ microparticles were collected and kept for further use.

The Pt atomic-level decoration was performed using a solution impregnation technique described elsewhere.^22^ The platinum precursor solution was prepared by dissolving 0.5 mM H_2_PtCl_6_ in a 50 vol % methanol solution, and bTiO_2_ microparticles were added to reach a concentration of 0.1 wt %. The theoretical Pt loading was ∼3 atom % with respect to bTiO_2_ microparticles. The reaction mixture was magnetically stirred for 24 h under dark conditions in a nitrogen atmosphere. The yielded Pt-bTiO_2_ microparticles were collected, washed in ethanol and distilled water three times, and dried at 60 °C overnight.

Magnetic NPs were synthesized according to a previously reported procedure.^51^ Decoration of the bTiO_2_ and Pt-bTiO_2_ microrobots was achieved by incubating the bTiO_2_ and Pt-bTiO_2_ microparticles with magnetic NPs in a 50 vol % ethanol solution for 5 h at room temperature using a shaker (130 rpm). The loading of magnetic NPs was set to 10 wt % with respect to the bTiO_2_ and Pt-bTiO_2_ microparticles; i.e., 10 mg of magnetic NPs was used to decorate 90 mg of bTiO_2_ or Pt-bTiO_2_ microrobots. The resulting microrobots were collected using a permanent magnet, washed with water three times, and dried at 60 °C overnight.

### Characterization

UV–vis spectroscopy was performed by using a UV/vis/NIR spectrophotometer (Jasco, V570) equipped with a PbS NIR detector. The diffuse reflectance was measured from powder samples by using an integrating sphere (ISN-470); the resulting spectra were processed into absorption spectra using the Spectra Manager software. Powder X-ray diffractograms were obtained with a MiniFlex X-ray diffractometer (XRD) (Rigaku) equipped with a Cu target and D/tex Ultra 2 detector. Morphology characterization was performed using a Hitachi S4800 scanning electron microscope (SEM). EDX mapping was performed using an EDS detector by Oxford Instruments coupled with an SEM (Hitachi SU8330). The samples for SEM characterization were prepared by drop-casting the corresponding colloidal solution onto a silicon substrate. Transmission electron microscopy (TEM) characterization was performed using a Talos F200X S/TEM microscope (ThermoFisher). The samples were prepared by dispersing them in an ethanol solution and subsequently drop-casting them on a STEM Cu grid (Cu150P, Okenshoji Co., Ltd.). XPS spectra of the samples deposited on a Si wafer were detected by using Quantera SXM (ULVAC-PHI) with an Al Kα X-ray source; the carbon correction was performed by setting the C 1*s* peak at 284.6 eV.

### Schooling Behavior

Schooling behavior and propulsion abilities of microrobots were evaluated using an optical microscope (Nikon Eclipse Ti2) equipped with a camera DSRi2. The light irradiation was from the bottom using an LED light source (Nikon, D-LEDI and UV source, pE-100, CoolLED) and filter cubes (UV: 360–370 nm, DAPI: 383–408 nm, FITC: 460–500 nm, mCherry: 550–590 nm, cy5:590–650 nm); the intensity of LED sources was set to 100%. The propulsion abilities were observed in an aqueous solution that was dropped onto a microscope glass slide. The propulsion abilities were monitored for 1 min under dark conditions (1–10 s), light irradiation (10–30 s), and dark conditions (30–60 s) by recording videos at 11 fps. The videos were subsequently processed by using Fiji software.

### Photocatalytic HER

Targeted microrobots were dispersed in a 10 vol % methanol solution at a concentration of 0.4 mg/mL using ultrasonication. The prepared reaction mixture was placed in a top-irradiation reaction cell equipped with a Pyrex window connected to a gas-tight circulation system in an amount of 120 mL. The reaction mixture was evacuated and filled with argon and subsequently irradiated from the top with a 300 W Xe lamp source (PerkinElmer, CERMAX PF300BF). The reaction mixture was externally stirred at 500 rpm without any stirring bar placed in the reaction mixture, allowing only the microrobot propulsion to perform the HER in the “on-the-fly” mode. For the control experiments that were performed in the static mode, the reaction mixture was not magnetically stirred. The progress of the HER was monitored using an online gas chromatograph (Shimadzu, GC-8A, MS-5A column, Ar carrier) coupled with a TCD detector (GC-TCD) for 5 h with sampling times at 0, 0.5, 1, 2, 3, 4, and 5 h. The H_2_ yield was calculated according to the calibration.

## Supplementary Material



## Notes

Additional electron microscopy micrographs and EDX elemental mapping of the TiO_2_ material and microrobots, additional spectra from EDX characterization of the bTiO_2_ material, control experiments of bTiO_2_ material as the photocatalyst, and structural characterization of photocatalysts after the HER (PDF)

## References

[1] Ang T. Z., Salem M., Kamarol M., Das H. S., Nazari M. A., Prabaharan N. (2022). A Comprehensive Study of Renewable Energy Sources:
Classifications, Challenges and Suggestions. Energy Strategy Rev..

[2] Melchionna M., Fornasiero P. (2020). Updates on
the Roadmap for Photocatalysis. ACS Catal..

[3] Wang Y., Wang T., Arandiyan H., Song G., Sun H., Sabri Y., Zhao C., Shao Z., Kawi S. (2024). Advancing
Catalysts by Stacking Fault Defects for Enhanced Hydrogen Production:
A Review. Adv. Mater..

[4] Tao X., Zhao Y., Wang S., Li C., Li R. (2022). Recent Advances
and Perspectives for Solar-Driven Water Splitting Using Particulate
Photocatalysts. Chem. Soc. Rev..

[5] Wu S., Salmon N., Li M. M.-J., Bañares-Alcántara R., Tsang S. C. E. (2022). Energy Decarbonization via Green H_2_ or NH_3_?. ACS Energy Lett..

[6] Gunawan D., Zhang J., Li Q., Toe C. Y., Scott J., Antonietti M., Guo J., Amal R. (2024). Materials
Advances
in Photocatalytic Solar Hydrogen Production: Integrating Systems and
Economics for a Sustainable Future. Adv. Mater..

[7] Kaiya K., Ueki Y., Kawamoto H., Watanabe K., Yoshino S., Yamaguchi Y., Kudo A. (2024). Water Splitting over Transition Metal-Doped
SrTiO_3_ Photocatalysts with Response to Visible Light up
to 660 nm. Chem. Sci..

[8] Ivanová L., Truksa J., Whang D. R., Sariciftci N. S., Yumusak C., Krajcovic J. (2024). Nature-Inspired Photocatalytic Hydrogen
Production with a Flavin Photosensitizer. ACS
Omega.

[9] Mai H., Le T. C., Chen D., Winkler D. A., Caruso R. A. (2022). Machine
Learning for Electrocatalyst and Photocatalyst Design and Discovery. Chem. Rev..

[10] Goh Z., Dolan A., de la Perrelle J. M., Jevric M., Pan X., Andersson M. R., Huang D. M., Kee T. W. (2024). Preparation-Dependent
Photocatalytic Hydrogen Evolution by Organic Semiconducting Nanoparticles. ACS Appl. Nano Mater..

[11] Ariga K. (2024). Nanoarchitectonics:
The Method for Everything in Materials Science. Bull. Chem. Soc. Jpn..

[12] Song J., Jancik-Prochazkova A., Kawakami K., Ariga K. (2024). Lateral Nanoarchitectonics
from Nano to Life: Ongoing Challenges in Interfacial Chemical Science. Chem. Sci..

[13] Ariga K., Li J., Fei J., Ji Q., Hill J. P. (2016). Nanoarchitectonics
for Dynamic Functional Materials from Atomic-/Molecular-Level Manipulation
to Macroscopic Action. Adv. Mater..

[14] Kaiser S. K., Chen Z., Faust Akl D., Mitchell S., Pérez-Ramírez J. (2020). Single-Atom
Catalysts across the Periodic Table. Chem. Rev..

[15] Wu S.-M., Schmuki P. (2025). Single Atom Cocatalysts
in Photocatalysis. Adv. Mater..

[16] Zhang Y., Zhao J., Wang H., Xiao B., Zhang W., Zhao X., Lv T., Thangamuthu M., Zhang J., Guo Y., Ma J., Lin L., Tang J., Huang R., Liu Q. (2022). Single-Atom Cu Anchored
Catalysts for Photocatalytic Renewable H_2_ Production with
a Quantum Efficiency of 56%. Nat. Commun..

[17] Qin S., Denisov N., Sarma B. B., Hwang I., Doronkin D. E., Tomanec O., Kment S., Schmuki P. (2022). Schmuki. Pt Single
Atoms on TiO_2_ Polymorphs – Minimum Loading with
a Maximized Photocatalytic Efficiency. Adv.
Mater. Interfaces.

[18] Wu Z., Hwang I., Cha G., Qin S., Tomanec O., Badura Z., Kment S., Zboril R., Schmuki P. (2022). Optimized
Pt Single Atom Harvesting on TiO_2_ Nanotubes – Towards
a Most Efficient Photocatalyst. Small.

[19] Zhong S., Shi H., Xiao C., Gu X., Wu J., Lu S., Yuan Z., Yang Y., Yu D., Chen X. (2025). Efficient
Photocatalytic Hydrogen Production via Single-Atom Pt Anchored Hydrogen-Bonded
Organic Frameworks. J. Colloid Interface Sci..

[20] Ju X., Pumera M. (2024). Single Atom Engineering
for Nanorobotics. ACS Nano.

[21] Jancik-Prochazkova A., Ariga K. (2025). Nano-/Microrobots for Environmental Remediation in the Eyes of Nanoarchitectonics:
Toward Engineering on a Single-Atomic Scale. Research.

[22] Jancik-Prochazkova A., Kmentova H., Ju X., Kment S., Zboril R., Pumera M. (2024). Precision Engineering of Nanorobots:
Toward Single
Atom Decoration and Defect Control for Enhanced Microplastic Capture. Adv. Funct. Mater..

[23] Xing Y., Xiu J., Zhou M., Xu T., Zhang M., Li H., Li X., Du X., Ma T., Zhang X. (2023). Copper Single-Atom
Jellyfish-like Nanomotors for Enhanced Tumor Penetratioin and Nanocatalytic
Therapy. ACS Nano.

[24] Soto F., Karshalev E., Zhang F., Esteban Fernandez de Avila B., Nourhani A., Wang J. (2022). Smart Materials for Microrobots. Chem. Rev..

[25] Kim J., Mayorga-Burrezo P., Song S.-J., Mayorga-Martinez C. C., Medina-Sanchez M., Pane S., Pumera M. (2024). Advanced Materials
for Micro/Nanorobotics. Chem. Soc. Rev..

[26] Ruiz-González N., Esporrín-Ubieto D., Kim I.-D., Wang J., Sánchez S. (2025). Micro- and Nanomotors: Engineered Tools for Targeted
and Efficient Biomedicine. ACS Nano.

[27] Dey K. K., Sen A. (2017). Chemically Propelled Molecules and
Machines. J. Am. Chem. Soc..

[28] Zhou H., Mayorga-Martinez C. C., Pane S., Zhang L., Pumera M. (2021). Magnetically
Driven Micro and Nanorobots. Chem. Rev..

[29] Zhao Y., Lin J., Wu Q., Ying Y., Puigmartí-Luis J., Pané S., Wang S. (2024). RevolutionizingRevolutionizing Tetracycline
Hydrochloride Remediation: 3D Motile Light-Driven MOFs Based Micromotors
in Harsh Saline EnvironmentsTetracycline Hydrochloride Remediation:
3D Motile Light-Driven MOFs Based Micromotors in Harsh Saline Environments. Adv. Sci..

[30] Torlakcik H., Sevim S., Alves P., Mattmann M., Llacer-Wintle J., Pinto M., Moreira R., Flouris A. D., Landers F. C., Chen X.-Z., Puigmartí-Luis J., Boehler Q., Mayor T. S., Kim M., Nelson B. J., Pané S. (2024). Magnetically
Guided Microcatheter for Targeted Injection of Magnetic Particle Swarms. Adv. Sci..

[31] Oral C. M., Pumera M. (2023). In Vivo Applications
of Micro/Nanorobots. Nanoscale.

[32] Urso M., Ussia M., Pumera M. (2023). Smart Micro- and Nanorobots for Water
Purification. Nat. Rev. Bioeng..

[33] Maria-Hormigos R., Mayorga-Martinez C. C., Kinčl T., Pumera M. (2023). Nanostructured Hybrid
BioBots for Beer Brewing. ACS Nano.

[34] Chen C., Ding S., Wang J. (2024). Materials Consideration
for the Design,
Fabrication and Operation of Microscale Robots. Nat. Rev. Mater..

[35] Cuntín-Abal C., Jurado-Sánchez B., Escarpa A. (2025). Micromotors for Antimicrobial
Resistance Bacteria Inactivation in Water Systems: Opportunities and
Challenges. Environ. Sci.: Nano.

[36] Ferrer
Campos R., Bachimanchi H., Volpe G., Villa K. (2023). Bubble-Propelled
Micromotors for Ammonia Generation. Nanoscale.

[37] Mallick A., Kim J., Pumera M. (2024). Magnetically Propelled Microrobots toward Photosynthesis
of Green Ammonia from Nitrates. Small.

[38] Ullattil S. G., Pumera M. (2023). Light-Powered Self-Adaptive Mesostructured Microrobots
for Simultaneous Microplastics Trapping and Fragmentation via in situ
Surface Morphing. Small.

[39] Ussia M., Urso M., Kment S., Fialova T., Klima K., Dolezelikova K., Pumera M. (2022). Light-Propelled Nanorobots for Facial
Titanium Implants Biofilms Removal. Small.

[40] Sahoo S. S., Mansingh S., Babu P., Parida K. (2021). Black Titania an Emerging
Photocatalyst: Review Highlighting the Synthesis Techniques and Photocatalytic
Activity for Hydrogen Generation. Nanoscale
Adv..

[41] Dai S., Wu Y., Sakai T., Du Z., Sakai H., Abe M. (2010). Preparation
of Highly Crystalline TiO_2_ Nanostructures by Acid-assisted
Hydrothermal Treatment of Hexagonal-structured Nanocrystalline Titania/Cetyltrimethylammonium
Bromide Nanoskeleton. Nanoscale Res. Lett..

[42] Yazaki D., Kawawaki T., Hirayama D., Kawachi M., Kato K., Oguchi S., Yamaguchi Y., Kikkawa S., Ueki Y., Hossain S., Osborn D. J., Ozaki F., Tanaka S., Yoshinobu J., Metha G. F., Yamazoe S., Kudo A., Yamakata A., Negishi Y. (2023). Carbon Nitride Loaded with an Ultrafine,
Monodisperse, Metallic Platinum-Cluster Cocatalyst for the Photocatalytic
Hydrogen-Evolution Reaction. Small.

[43] Kim D. W., Wrede P., Rodriguez-Camargo A., Chen Y., Dogan N. O., Glück C., Lotsch B. V., Razansky D., Sitti M. (2025). Upconversion
Nanoparticle-Covalent Organic Framework Core-Shell Particles as Therapeutic
Microrobots Trackable with Optoacoustic Imaging. Adv. Mater..

[44] Lv K., Hou M., Kou Y., Yu H., Liu M., Zhao T., Shen J., Huang X., Zhang J., Mady M. F., Elzatahry A. A., Li X., Zhao D. (2024). Black Titania Janus
Mesoporous Nanomotor for Enhanced Tumor Penetration and Near-Infrared
Light-Triggered Photodynamic Therapy. ACS Nano.

[45] Mou F., Zhang J., Wu Z., Du S., Zhang Z., Xu L., Guan J. (2019). Phototactic Flocking of Photochemical Micromotors. iScience.

[46] Zhang J., Mou F., Wu Z., Song J., Kauffman J. E., Sen A., Guan J. (2021). Cooperative
Transport by Flocking Phototactic Micromotors. Nanoscale Adv..

[47] Jiang S., Hao B., Song X., Jiang Y., Guo J., Wang Y., Wang Q., Wang X., Xu T., Wu X., Chan K. F., Chiu P. W. Y., Zhang L. (2025). Living Microalgae-Based
Magnetic Microrobots for Calcium Overload and Photodynamic Synergetic
Cancer Therapy. Adv. Healthcare Mater..

[48] Jancik-Prochazkova A., Jašek V., Figalla S., Pumera M. (2023). Photocatalytic Microplastics
“On-The-Fly” Degradation via Motile Quantum Materials-Based
Microrobots. Adv. Opt. Mater..

[49] Mayorga-Burrezo P., Mayorga-Martinez C. C., Pumera M. (2023). Photocatalysis Dramatically Influences
Motion of Magnetic Microrobots: Application to Removal of Microplastics
and Dyes. J. Colloid Interface Sci..

[50] De
la Asunción-Nadal V., Solano E., Jurado-Sánchez B., Escarpa A. (2024). Photophoretic MoS2-Fe2O3 Piranha Micromotors for Collective
Dynamic Microplastics Removal. ACS Appl. Mater.
Interfaces.

[51] Jancik-Prochazkova A., Jancik J., Palacios-Corella M., Pumera M. (2024). Microrobots Enhancing
Synthetic Chemistry Reactions in Non-Aqueous Media. Adv. Funct. Mater..

[52] Urso M., Ussia M., Novotný F., Pumera M. (2022). Trapping and Detecting
Nanoplastics by MXene-Derived Oxide Microrobots. Nat. Commun..

[53] He Y., Liu Y., Shang W., Tan P. (2025). Hydrogen Bubble Evolution and Its
Induced Mass Transfer on Zinc Electrodes in Alkaline and Neutral Media. Nanoscale.

[54] Markovskaya D. V., Cherepanova S. V., Gerasimov E. Y., Zhurenok A. V., Selivanova A. V., Selishchev D. S., Kozlova E. A. (2020). The Influence of the Sacrifical Agent
Nature on Transformations of the Zn­(OH)_2_/Cd_0.3_Zn_0.7_S Photocatalyst During Hydrogen Production under
Visible Light. RSC Adv..

[55] Sinhamahapatra A., Jeon J.-P., Yu J.-S. (2015). A New Approach to Prepare Highly
Active and Stable Black Titania for Visible Light-Assisted Hydrogen
Production. Energy Environ. Sci..

[56] Urso M., Ussia M., Peng X., Oral C. M., Pumera M. (2023). Reconfigurable
Self-Assembly of Photocatalytic Magnetic Microrobots for Water Purification. Nat. Commun..

[57] Guo J., Gao B., Li Q., Wang S., Shang Y., Duan X., Xu X. (2024). Size-Dependent
Catalysis in Fenton-like Chemistry: From Nanoparticles
to Single Atoms. Adv. Mater..

[58] Shi X., Dai C., Wang X., Hu J., Zhang J., Zheng L., Mao L., Zheng H., Zhu M. (2022). Protruding Pt Single-Sites on Hexagonal
ZnIn_2_S_4_ to Accelerate Photocatalytic Hydrogen
Evolution. Nat. Commun..

[59] Zhang Q., Yue M., Chen P., Ren Q., Kong W., Jia C., Lu Q., Wu J., Li Y., Liu W., Li P., Fu Y., Ma J. (2024). Accelerating Photocatalytic Hydrogen Production by
Anchoring Pt Single Atoms on Few-Layer g-C_3_N_4_ Nanosheets with Pt-N Coordination. J. Mater.
Chem. C.

[60] Liu L., Chen T., Chen Z. (2024). Understanding
the Dynamic Aggregation
in Single-Atom Catalysis. Adv. Sci..

[61] DeRita L., Resasco J., Dai S., Boubnov A., Thang H. V., Hoffman A. S., Ro I., Graham G. W., Bare S. R., Pacchioni G., Pan X., Christopher P. (2019). Structural
Evolution of Atomically Dispersed Pt Catalysts Dictates Reactivity. Nat. Mater..

[62] Hejazi S., Mohajernia S., Osuagwu B., Zoppellaro G., Andryskova P., Tomanec O., Kment S., Zbořil R., Schmuki P. (2020). On the Controlled Loading of Single Platinum Atoms
as a Co-Catalyst on TiO_2_ Anatase for Optimized Photocatalytic
H_2_ Generation. Adv. Mater..

[63] Lv C., Wang T., Fang Y., Ying Y., Ji L., Liu L., Wang S. (2025). Pt Single Atom and Atomic Cluster-Enhanced TiO_2_ Janus Micromotors for Efficient Bubble Propulsion and Photocatalytic
Environmental Remediation. Inorg. Chem. Front..

[64] Chen X., Liu L., Yu P. Y., Mao S. S. (2011). Increasing Solar Absorption for Photocatalysis
with Black Hydrogenated Titanium Dioxide Nanocrystals. Science.

[65] Zhang J., Xu Q., Feng Z., Li M., Li C. (2008). Importance of the Relationship
between Surface Phases and Photocatalytic Activity of TiO_2_. Angew. Chem., Int. Ed..

[66] Hou W., Cronin S. B. (2013). A Review of Surface Plasmon Resonance-Enhanced Photocatalysis. Adv. Funct. Mater..

[67] Li Y., Zhang L., Fan Y., Cheng H., Li F. (2016). Plasmon-Enhanced
Photocatalysis on Pt–TiO_2_ Nanocomposites: Tuning
Photoresponse through Pt Nanoparticle Deposition. J. Mater. Chem. A.

[68] Ma T., Li W., Li J., Duan W., Gao F., Liao G., Li J., Wang C. (2024). Multisite Coccatalysis: Single Atomic Pt^2+^/Pt^0^ Active Sites Synergistically Improve the Simulated
Sunlight Driven H_2_O-to-H_2_ Conversion Performance
of Sb_2_S_3_ Nanorods. J.
Colloid Interface Sci..

