# Supplementary material for: Motion-Controlled Photocatalytic Hydrogen Evolution Using Microrobots Designed with a Single Atomic-Level Precision
Source: J Am Chem Soc. 2025 Jun 13;147(25):22003–14. doi: 10.1021/jacs.5c05661 (PMC12203592; doi:10.1021/jacs.5c05661)
Supplement: Supplementary file 1 [file ja5c05661_si_001.pdf]

# SUPPORTING INFORMATION

# Motion Controlled Photocatalytic Hydrogen Evolution Using Microrobots Designed with a Single Atomic-Level Precision

Anna Jancik-Prochazkova,*[a] Riku Nakao,[b] Yuichi Yamaguchi,[b,c] Akihiko Kudo,[b,c] Katsuhiko Ariga[a,d]

[a]Research Center for Materials Nanoarchitectonics, National Institute for Materials Science (NIMS), 1-1 Namiki, Tsukuba 305-0044, Japan

[b]Department of Applied Chemistry, Faculty of Science, Tokyo University of Science, 1-3 Kagurazaka, Shinjuku-ku, Tokyo 162-8601, Japan

[c]Carbon Value Research Center, Research Institute for Science and Technology, Tokyo University of Science, 2641 Yamazaki Noda-Shi, Chiba-ken 278-8510, Japan

[d]Graduate School of Frontier Sciences, The University of Tokyo, 5-1-5 Kashiwa-no-ha Kashiwa 277-8561, Japan

***E-mail:** jancik.prochazkovaanna@nims.go.jp

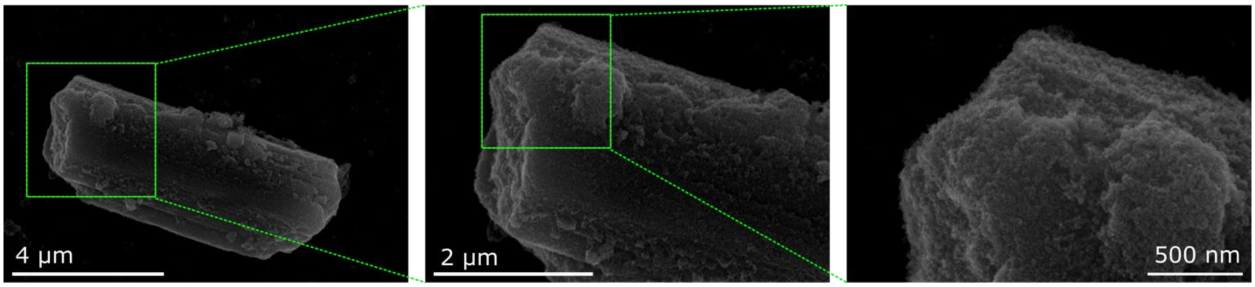


**Fig. S1.** SEM micrographs of pristine $TiO_2$ microparticles prepared via hydrothermal method.

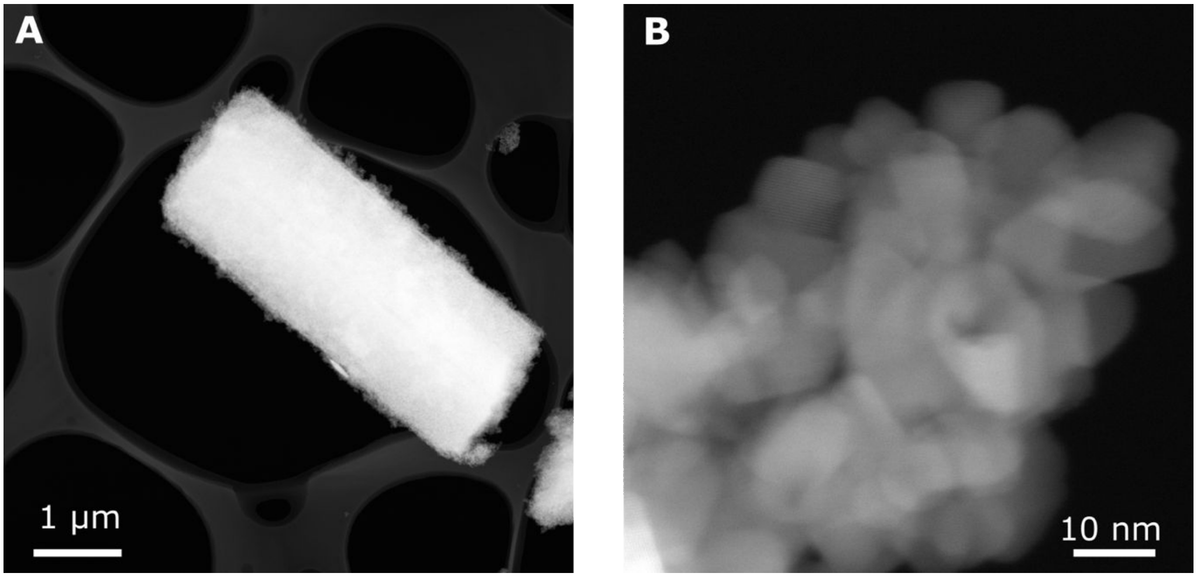


**Fig. S2.** STEM of a $bTiO_2$ microrod. A) Morphology of a representative microrod. B) Detail of the surface morphology.

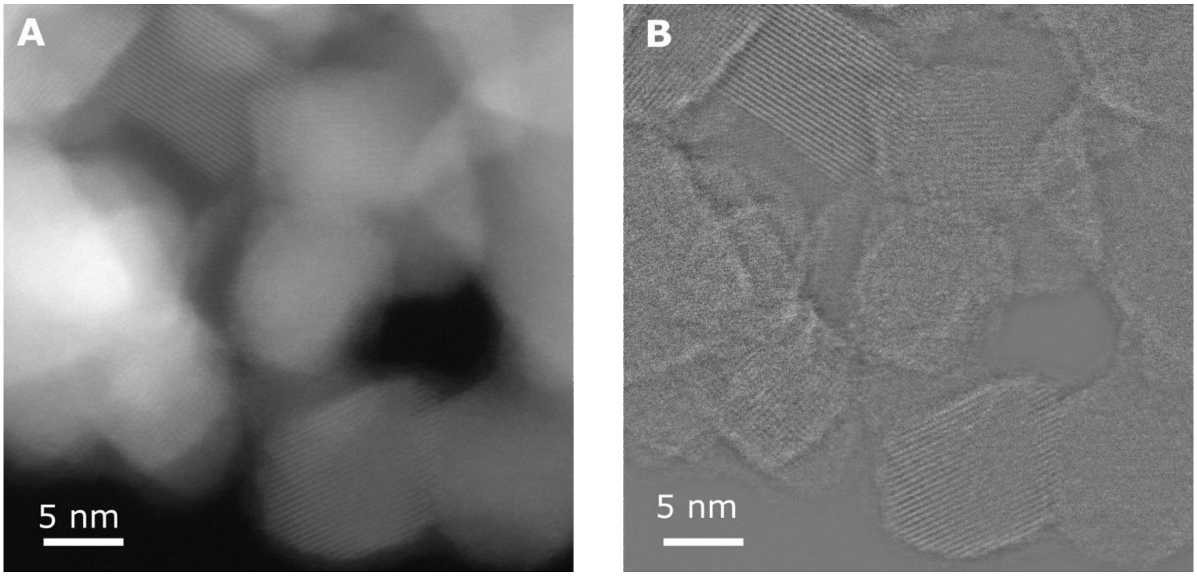


**Fig. S3.** STEM of a $bTiO_2$ microrod. A) An original STEM micrograph with detailed surface morphology and B) the micrograph after applying high-pass filter. The micrograph is a duplicate of Fig. 2D without any labels and modifications.

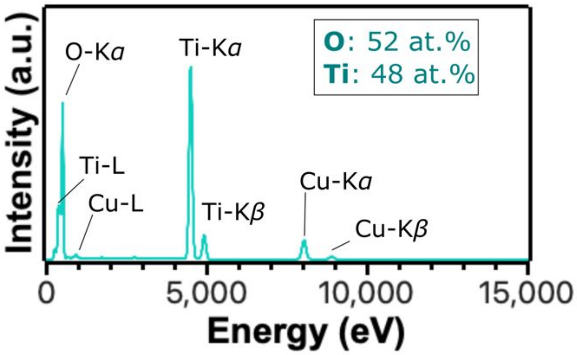


**Fig. S4.** EDX spectrum of $bTiO_2$ microrod demonstrating the presence of Ti and O. The signal of Cu is assigned to the signal of the STEM grid that was used as a substrate for sample deposition.

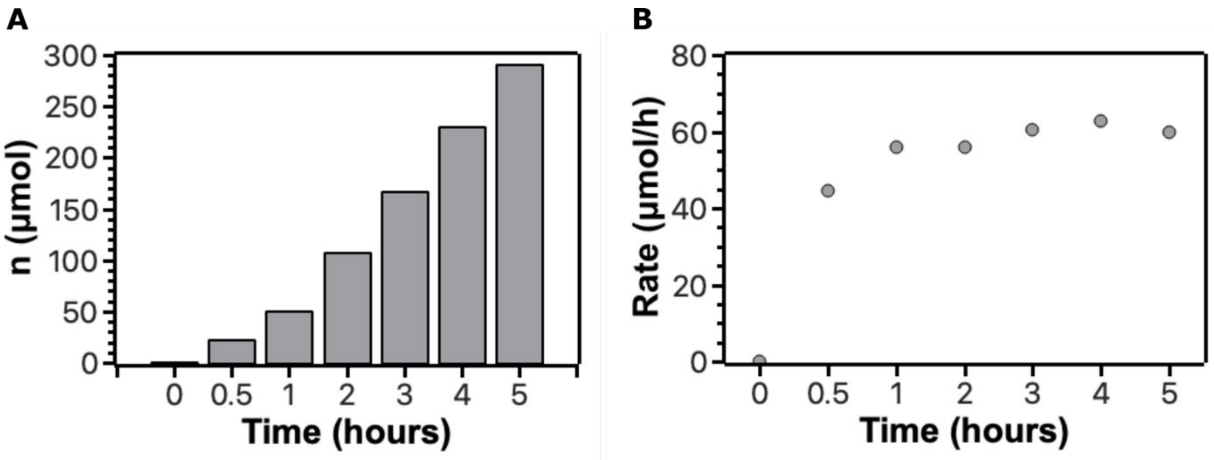


**Fig. S5.** Results of HER using $bTiO_2$ microparticles as the photocatalyst in the traditional photocatalytic HER setting using a magnetic stirring bar without considering only the contribution of the autonomous motion of microrobots. A) Yield of $H_2$ over time and B) reaction rate of $H_2$ generation.

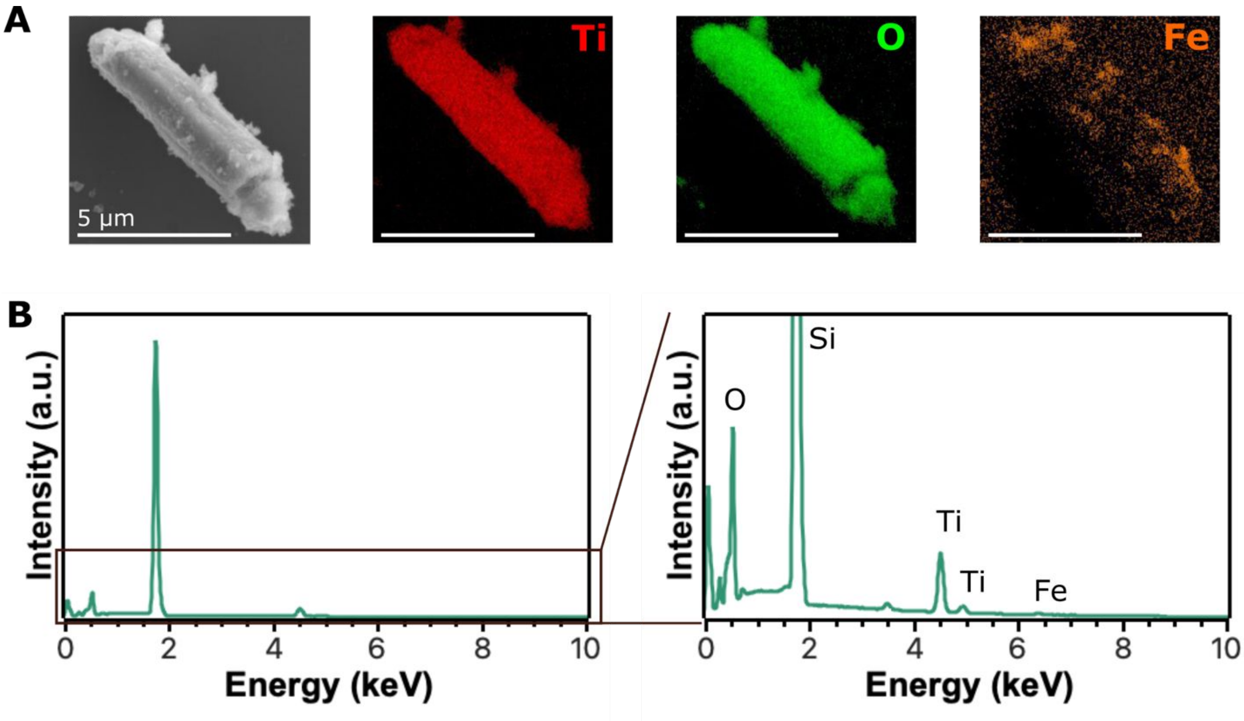


**Fig. S6.** EDX analysis of mag-$bTiO_2$ microrobots. A) Micrographs from elemental mapping, the scale bar is of 5 µm for all images. B) Spectra demonstrating the presence of Ti, O, and Fe. The signal of Si origins from the Si wafer that was used as a substrate for the deposition of the sample.

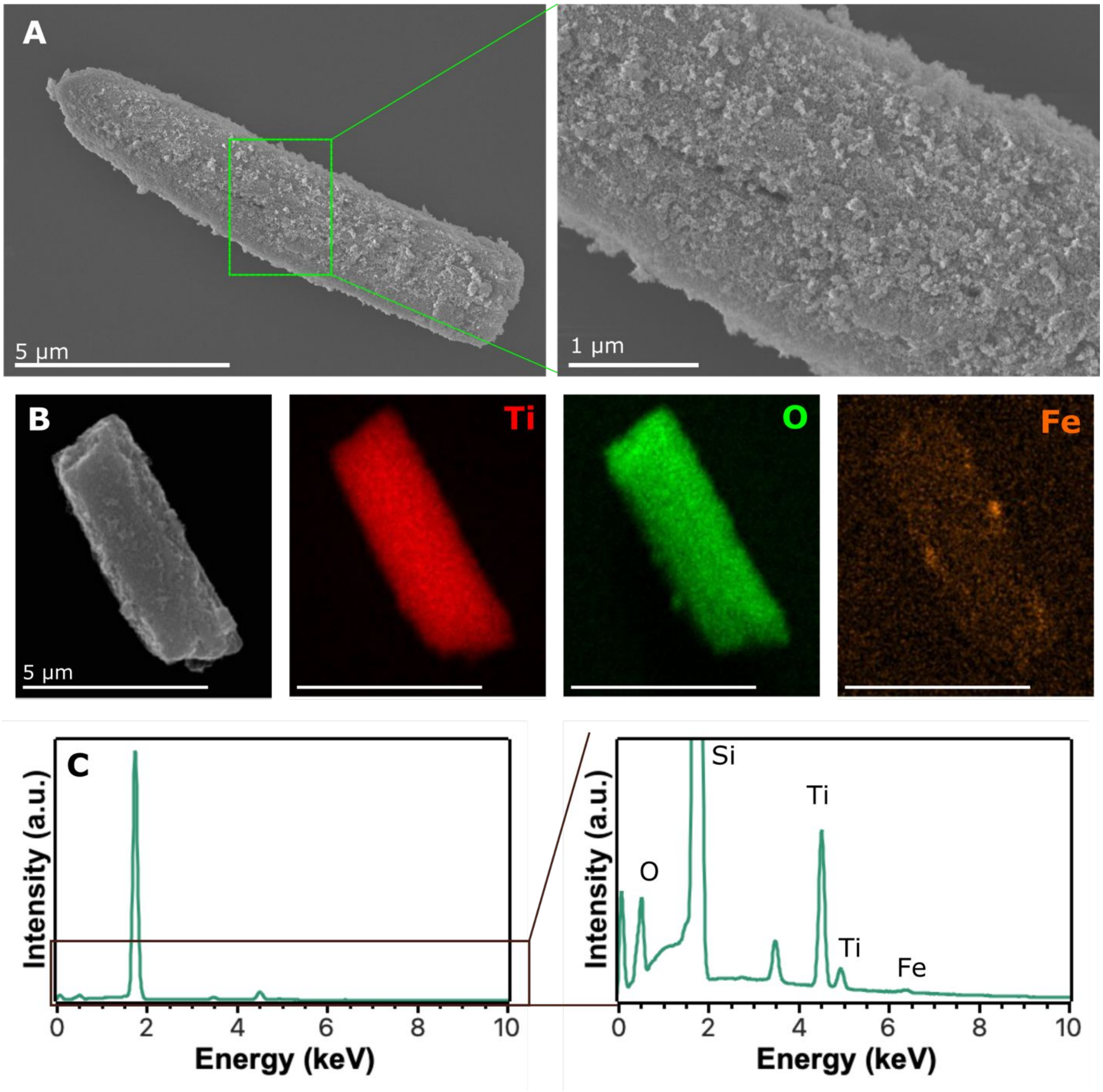


**Fig. S7.** Structural characterization of mag-b$TiO_2$ microrobots after completing HER. A) SEM micrograph of a representative microrobot collected after HER. B) Micrographs from elemental mapping, the scale bar is of 5 μm for all images. C) EDX spectra demonstrating the presence of Ti, O, and Fe. The signal of Si origins from the Si wafer that was used as a substrate for the sample preparation.

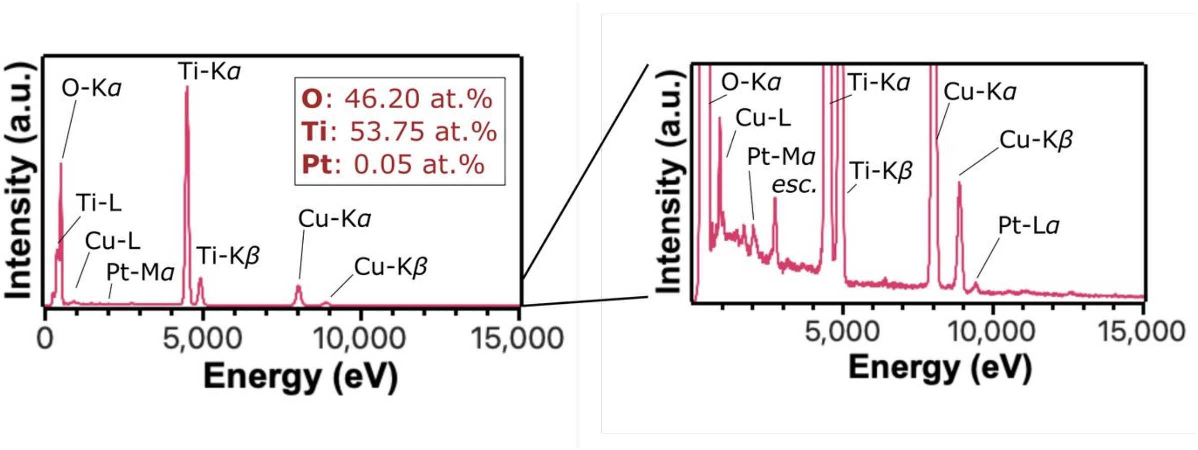


**Fig. S8.** EDX spectrum of STEM characterization of Pt-b$TiO_2$ microrobots. The signal of Cu is assigned to the signal of the STEM grid that was used as a substrate for sample deposition. Esc. is an abbreviation used for the escape peak of Ti.

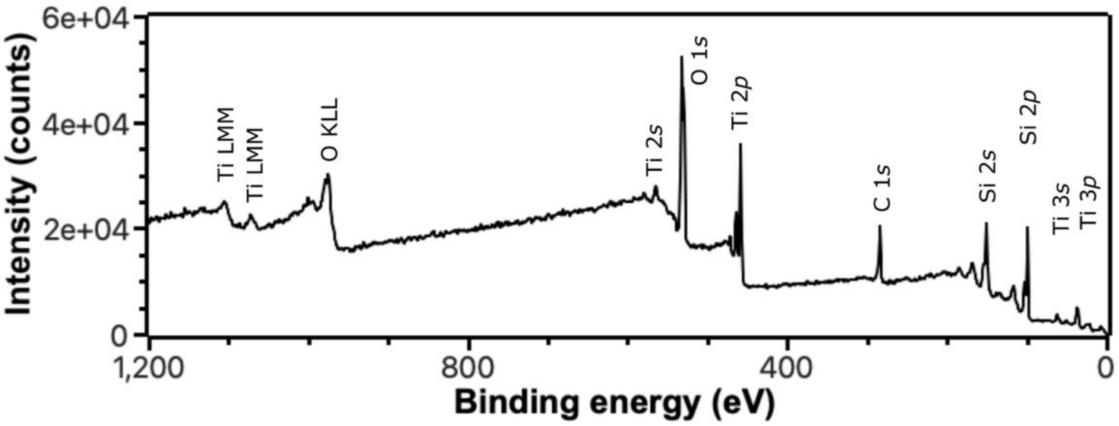


**Fig. S9.** XPS survey spectrum of the Pt-b$TiO_2$ sample. The signal of Pt is not visible in the broad spectrum.

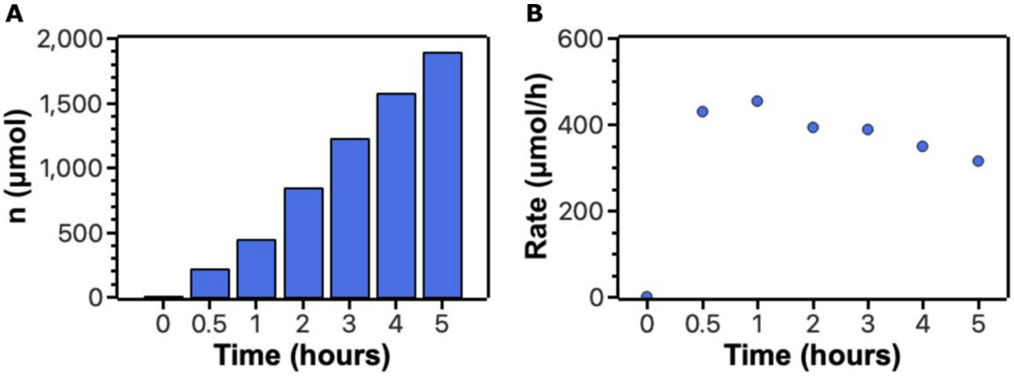


**Fig. S10.** Results of HER using Pt-b$TiO_2$ microparticles as photocatalysts in the traditional photocatalytic HER setting using a magnetic stirring bar without considering only the contribution of the autonomous motion of microrobots. A) Yield of $H_2$ over time and B) reaction rate of $H_2$ generation

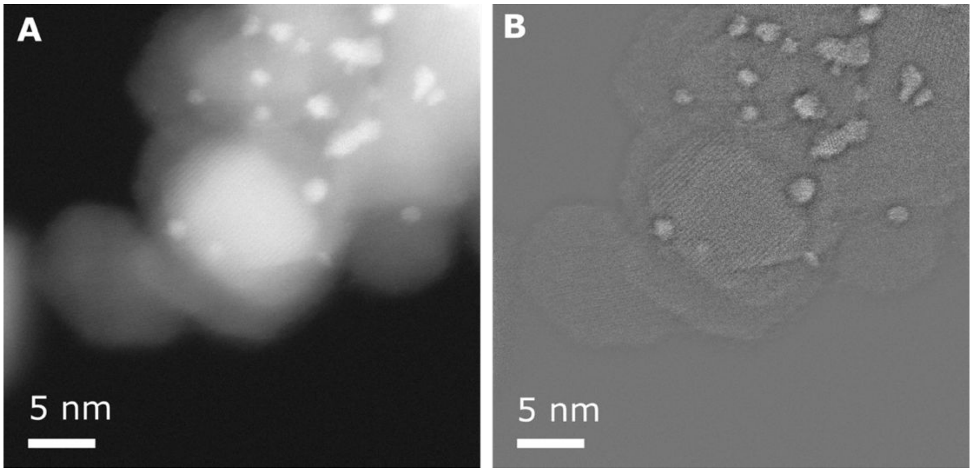


**Fig. S11.** STEM-HAADF characterization of Pt-b$TiO_2$ microparticles after HER. A) A micrograph demonstrating the presence of Pt clusters and B) the same micrograph after applying the high-pass filter.

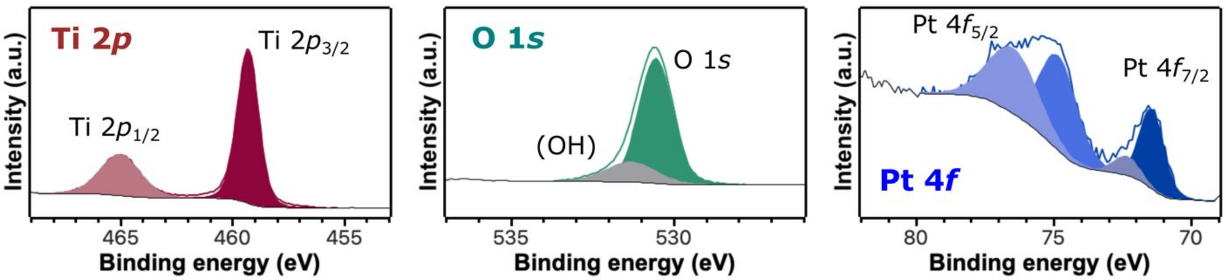


**Fig. S12.** XPS of Pt-b$TiO_2$ photocatalyst after HER. High resolution spectra of Ti, O, and Pt.

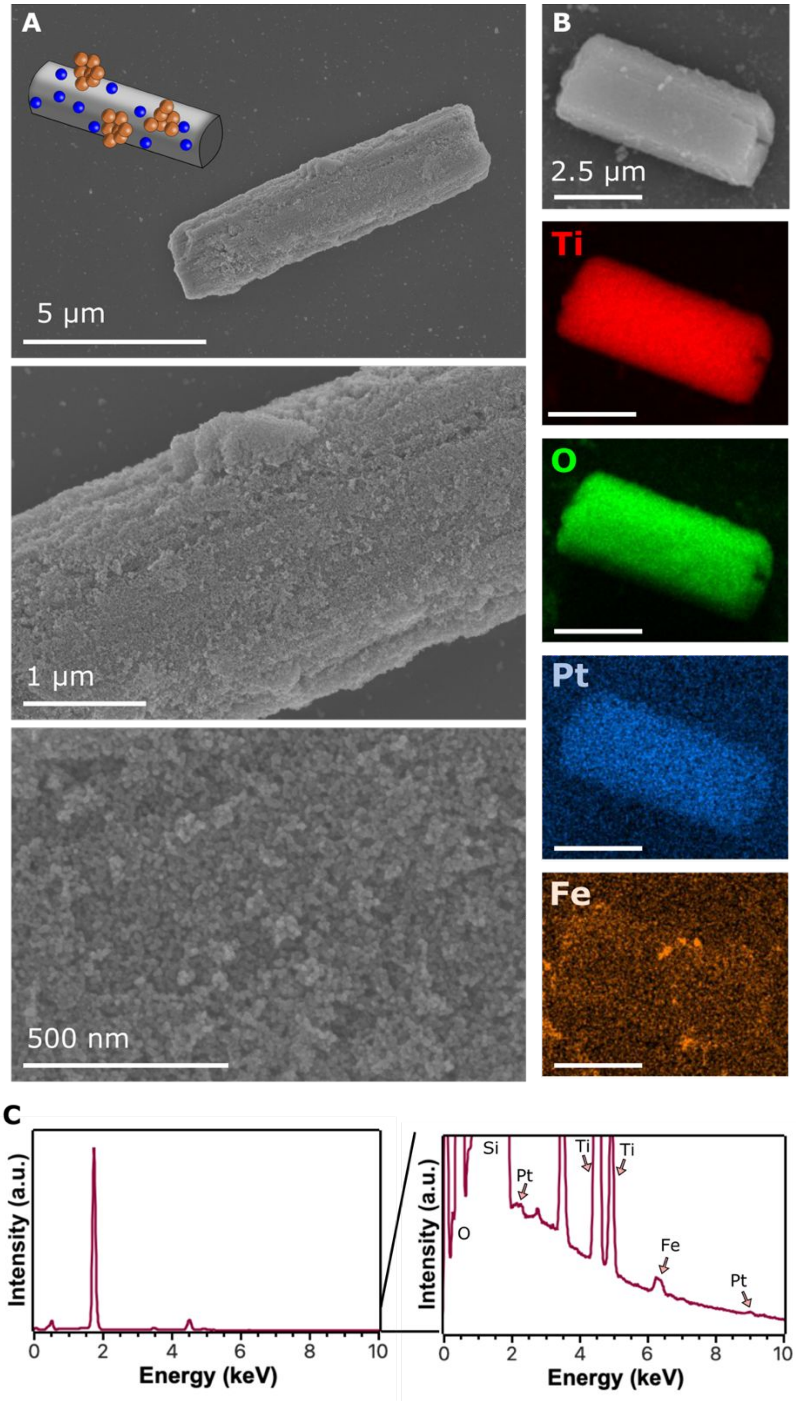


**Fig. S13.** Structural characterization of mag-Pt-b$TiO_2$ microrobots. A) SEM micrographs of a representative microrobot with zoomed details. B) Elemental mapping using EDS, the scale bar is of 2.5 μm in all micrographs. C) EDX spectra demonstrating the presence of Ti, O, Pt, and Fe. The signal of Si origins from the Si wafer that was used as a substrate for the sample preparation.

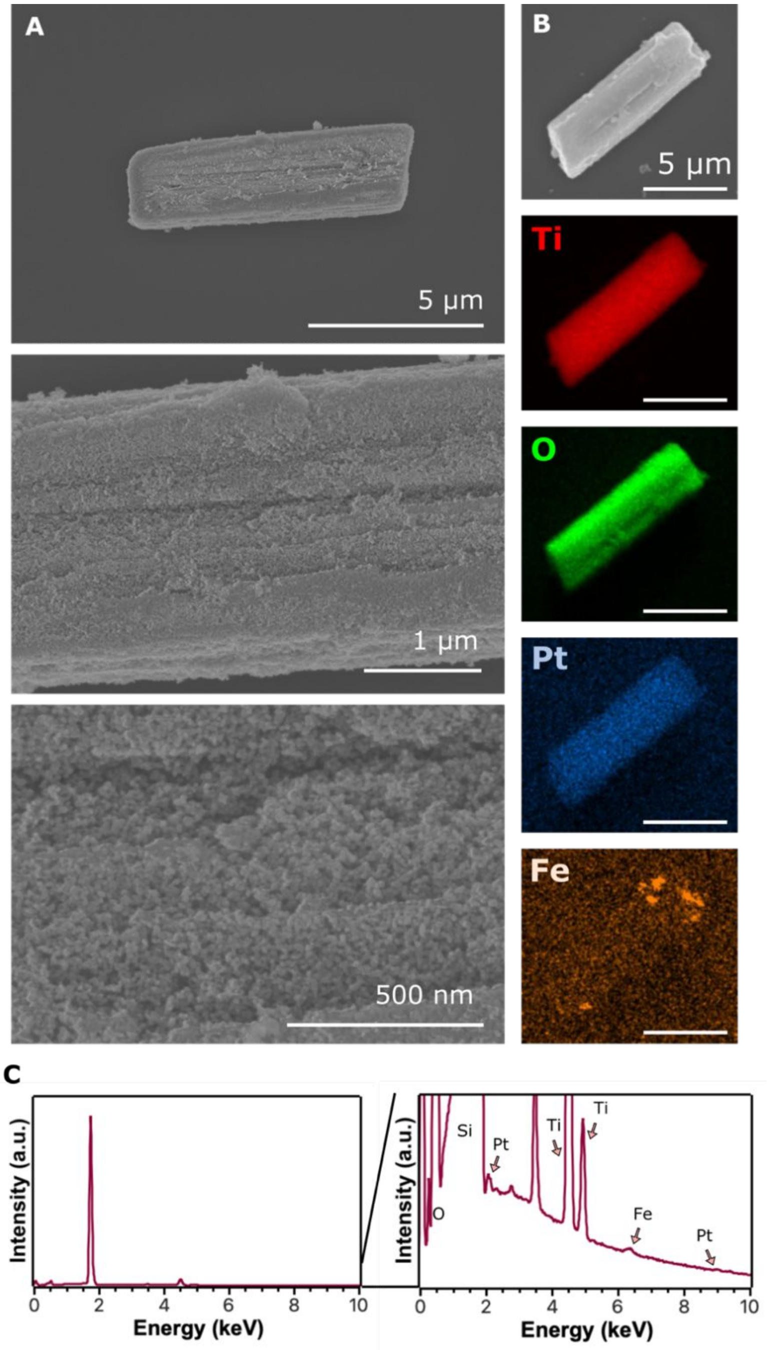


**Fig. S14.** Structural characterization of mag-Pt-b$TiO_2$ microrobots isolated after completing HER. A) SEM micrographs of a representative microrobot with zoomed details. B) Elemental mapping using EDS, the scale bar is of 5 μm in all micrographs. C) EDX spectra demonstrating the presence of Ti, O, Pt, and Fe. The signal of Si origins from the Si wafer that was used as a substrate for the sample preparation.
